# Post-Translational Modifications in Cancer-Associated CD8⁺ T-Cell Exhaustion: Mechanisms and Therapeutic Opportunities

**DOI:** 10.7150/ijbs.138638

**Published:** 2026-07-30

**Authors:** Yongqi Lv, Qihang Shang, Yang Yu, Yahui Fu, Lijuan Liu, Changgang Sun

**Affiliations:** 1 College of First Clinical Medicine, Shandong University of Traditional Chinese Medicine, Jinan, Shandong, 250000, China.; 2 Faculty of Chinese Medicine and State Key Laboratory of Mechanism and Quality of Chinese Medicine, Macau University of Science and Technology, Macau, 999078, China.; 3 Department of Oncology, Weifang Traditional Chinese Hospital, Weifang, Shandong, 261000, China.; 4 College of Traditional Chinese Medicine, Shandong Second Medical University, Weifang, Shandong, 261000, China.

**Keywords:** cancer immunotherapy, CD8⁺ T-cell exhaustion, post-translational modifications, tumor microenvironment, immune checkpoint blockade

## Abstract

CD8⁺ T-cell exhaustion is a distinct differentiation state driven by persistent antigen stimulation, and its establishment and maintenance are major barriers to effective cancer immunotherapy. Although the transcriptional and epigenetic landscapes of exhausted CD8⁺ T cells have been extensively characterized, it remains unclear how sustained external stimulation is integrated at the level of protein function to produce stable dysfunction and altered cell fate. Post-translational modifications constitute a key regulatory layer of protein function. They form a dynamic network that links persistent antigenic stimulation and tumor microenvironmental stress to cell fate, and therefore provide a critical entry point for understanding how exhaustion is initiated and maintained. In this review, we focus on how post-translational modifications convert persistent antigen stimulation and tumor microenvironmental stress into protein-level dysregulation and ultimately lock CD8⁺ T cells into an exhausted fate. We summarize how multiple classes of post-translational modifications drive exhaustion through effects on signal transduction, protein homeostasis, metabolic stress responses, and epigenetic reprogramming. We then discuss potential intervention strategies centered on critical regulatory nodes that may preserve the plasticity of precursor exhausted CD8⁺ T cells, restrain stabilization of the terminally exhausted state in CD8⁺ T cells, and optimize rational combination therapies. Finally, we outline the translational challenges and future directions of targeting post-translational modifications, and emphasize that identifying actionable modification nodes will be important for patient stratification and combination design in cancer immunotherapy.

## 1. Introduction

Immunotherapies such as immune checkpoint inhibitors (ICIs) have reshaped the therapeutic landscape of cancer, yet the overall response rate remains limited 1. Functional impairment of CD8⁺ T cells in the tumor microenvironment (TME) is now recognized as one of the main factors restricting therapeutic efficacy 1, 2. A central manifestation of this dysfunctional state is CD8⁺ T-cell exhaustion (Tex). The establishment and maintenance of CD8⁺ Tex are jointly driven by persistent antigen stimulation, suppressive cues in the TME, and metabolic stress 2. Hallmarks of this state include reduced proliferative capacity, diminished production of interleukin-2 (IL-2), interferon-γ (IFN-γ), and tumor necrosis factor-α (TNF-α), sustained upregulation of inhibitory receptors such as programmed cell death protein 1 (PD-1), cytotoxic T-lymphocyte-associated protein 4 (CTLA-4), T-cell immunoglobulin and mucin-domain-containing protein 3 (TIM-3), and lymphocyte activation gene-3 (LAG-3), and broad metabolic and epigenetic reprogramming 2. These changes do not occur in isolation. Instead, they form an integrated functional network that drives and stabilizes exhaustion, thereby limiting effector function and durability.

Exhausted CD8⁺ T cells display relatively stable molecular and epigenetic features. Even after blockade of the PD-1/programmed death-ligand 1 (PD-L1) axis, functional restoration is often incomplete and difficult to sustain 3. This observation suggests the presence of a relatively stable "epigenetic scar" in CD8⁺ Tex, which is widely considered a key basis for the stable maintenance of exhaustion and resistance to immunotherapy 3, 4. In recent years, substantial progress has been made in defining the transcriptional programs, epigenetic landscapes, and lineage heterogeneity of CD8⁺ Tex. However, these studies have largely described state features rather than fully explaining how exhaustion is established and durably maintained. In particular, how persistent antigen stimulation, suppressive signals within the TME, and metabolic pressure are continuously integrated by CD8⁺ T cells and converted into stable dysfunction and altered cell fate remains a central mechanistic question.

Dynamic changes in protein activity, stability, subcellular localization, and molecular interactions represent a critical layer linking external stimulation to long-term fate fixation 5. Post-translational modifications (PTMs) are one of the major regulatory mechanisms operating at this layer. These include phosphorylation, ubiquitination, acetylation, methylation, lactylation, and other dynamic covalent modifications. With the rapid development of mass spectrometry-based proteomics and chemical proteomics, the systematic identification, quantification, and functional interrogation of PTM sites have advanced considerably 6. Increasing evidence indicates that PTMs are not merely accompanying events during the development of CD8⁺ Tex. Rather, they constitute a major regulatory layer that governs exhaustion initiation, maintenance, and terminal stabilization. By remodeling signaling pathways, maintaining the homeostasis of checkpoint proteins and other critical factors, regulating transcription factor activity and nuclear dynamics, reprogramming metabolic stress and mitochondrial homeostasis, and shaping epigenetic states, PTMs continuously influence the differentiation trajectory of exhausted CD8⁺ T cells 7. However, these mechanisms still require systematic integration.

In this review, we therefore examine four major functional layers, including signal transduction, protein homeostasis, metabolic stress responses, and epigenetic states, to summarize how distinct PTM classes regulate the formation, maintenance, and terminal stabilization of CD8⁺ Tex. We further discuss their potential translational value in preserving the precursor exhausted T-cell (Tpex) pool, limiting stabilization of the terminally exhausted state (Tex^term^), and improving the efficacy of immune checkpoint blockade (ICB), adoptive cell therapy (ACT), radiotherapy, and chemotherapy.

## 2. Hierarchical differentiation of exhausted CD8⁺ T cells

The concept of "T-cell exhaustion" was first established in models of chronic lymphocytic choriomeningitis virus (LCMV) infection 8. During acute viral infection, naive T cells become activated after antigen recognition and co-stimulation, differentiate into effector T cells, exert cytotoxic activity, and produce effector cytokines 9. Under conditions of persistent antigen stimulation, however, CD8⁺ T cells progressively deviate from the canonical effector differentiation trajectory and enter an exhausted state. Cytokine production is lost in a characteristic hierarchical manner: IL-2 is impaired first, followed by TNF-α, whereas IFN-γ is further attenuated under sustained stimulation, reflecting the progressive deepening of CD8⁺ Tex 10.

With the development of tumor immunology and single-cell studies, the hierarchical differentiation framework revealed by chronic infection models has been further extended to the tumor context. Studies using mouse tumor models and human tumor samples have shown that tumor-infiltrating CD8⁺ T cells also contain transcriptional and functional states corresponding to precursor-like, intermediate, and terminal exhaustion, which are associated with clonal expansion and therapeutic responses after immune checkpoint blockade 11-14. However, cancer-associated CD8⁺ Tex are not simply equivalent to Tex induced by chronic infection. Instead, they constitute a tumor-contextualized exhaustion continuum shaped by persistent tumor antigen/neoantigen exposure, checkpoint ligand signaling, and immunosuppressive and metabolic pressures within the TME 15, 16.

**Figure 1:**
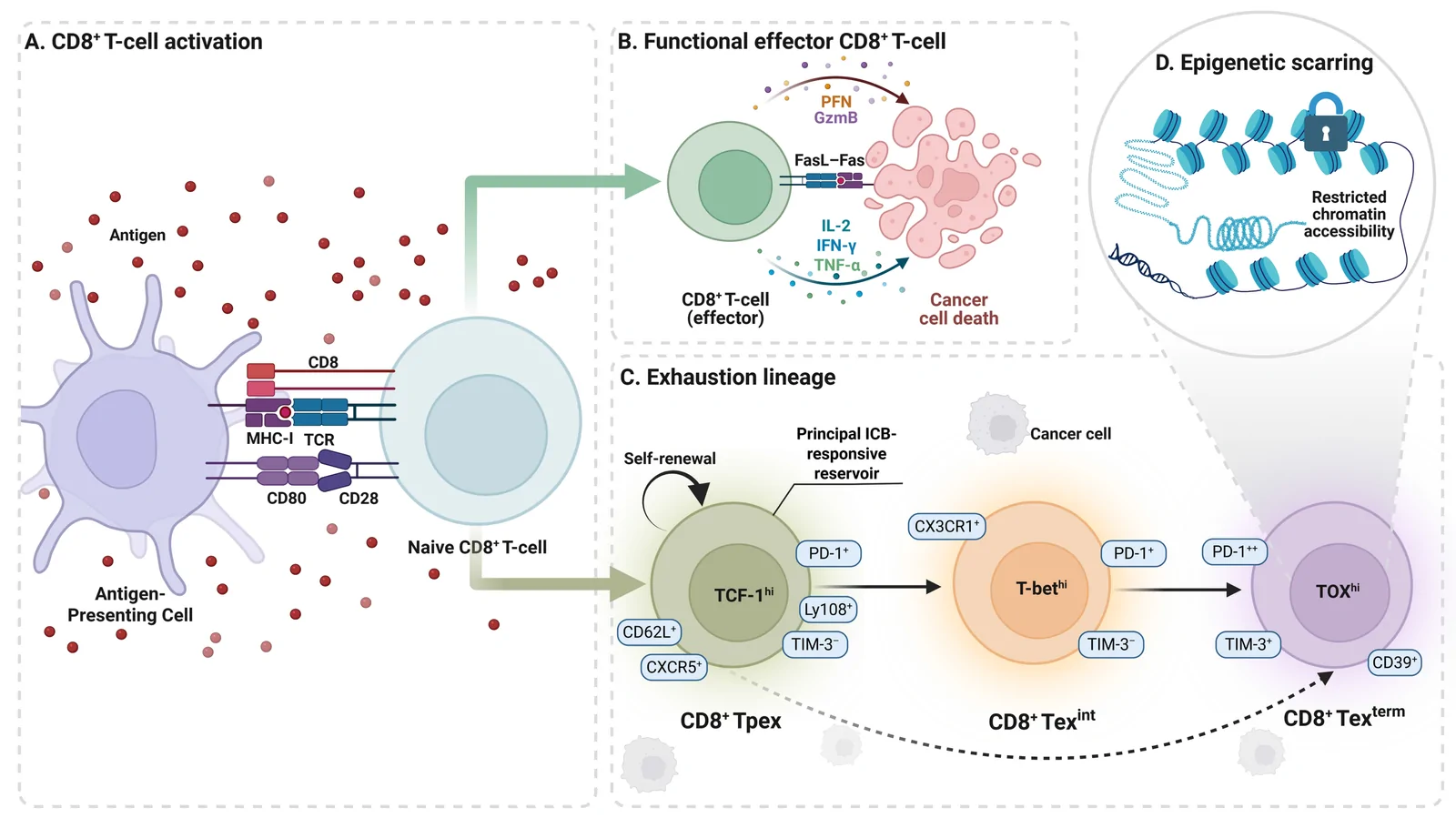
Activation, effector differentiation, exhaustion lineage progression, and epigenetic scar formation in CD8⁺ T cells. (A) Naïve CD8⁺ T cells are activated by antigen-presenting cells. (B) Functional effector CD8⁺ T cells mediate antitumor activity through the release of perforin (PFN) and granzyme B (GzmB), FasL-Fas-mediated cytotoxicity, and effector cytokine production. (C) Under persistent antigen stimulation, cells progressively differentiate along the exhaustion lineage. TCF-1^hi^ precursor exhausted T cells (Tpex), which retain self-renewal capacity and form the main reservoir that responds to immune checkpoint blockade (ICB), transition into a T-bet^hi^ intermediate exhausted state (Tex^int^) and then into a TOX^hi^ terminally exhausted state (Tex^term^), accompanied by dynamic changes in inhibitory receptors and differentiation markers. (D) Terminal exhaustion is associated with the formation of an "epigenetic scar", characterized by restricted chromatin accessibility, indicating that the exhausted fate has been stably fixed and is only partially reversible.

In this tumor context, exhausted CD8⁺ T cells can generally be further divided into Tpex, intermediate exhausted cells (Tex^int^), and highly differentiated Tex^term^ 17. Tpex are thought to constitute the reservoir population of the exhausted lineage and retain self-renewal and regenerative capacity. They typically express high levels of T-cell factor 1 (TCF-1), relatively low levels of PD-1, and frequently co-express surface markers such as signaling lymphocytic activation molecule family member 6 (SLAMF6), L-selectin (CD62L), and C-X-C motif chemokine receptor 5 (CXCR5) 18. The proliferative burst observed after PD-1 blockade arises mainly from this precursor-like population 19. CD8⁺ T cells lacking TCF-1 fail to survive long term and respond poorly to anti-PD-L1 therapy 20. As TCF-1 expression declines, cells may transition into Tex^int^, a state characterized by high T-bet expression and partial effector-like transcriptional features, which serves as an intermediate stage between Tpex and terminal exhaustion 21. Tex^term^ may arise from Tex^int^ through TOX-mediated antagonism of TBX21 (T-bet), leading to T-bet loss, or may develop directly from Tpex under TCF-1-dependent regulation. This population displays low proliferative activity, severely impaired effector function, and sustained high expression of inhibitory receptors and TOX 21. At the epigenetic level, Tex^term^ cells exhibit a chromatin landscape distinct from that of effector and memory T cells and acquire a relatively stable "epigenetic scar". This scar is primarily characterized by the persistent retention of an exhaustion-associated epigenetic landscape, involving alterations in chromatin accessibility, DNA methylation, histone modifications, and chromatin remodeling. By maintaining exhaustion-associated transcriptional programs and restricting the reactivation of effector- and memory-associated gene programs, these alterations promote Tex^term^ fate stabilization and limit the functional reversibility of Tex^term^ cells 3, 22-24 (Fig. 1).

This dynamic differentiation trajectory from Tpex through Tex^int^ to Tex^term^ highlights why PTMs are central to fate determination in CD8⁺ Tex. Whether CD8⁺ T cells retain Tpex-like plasticity or progress toward Tex^term^ is not dictated solely by a given gene-expression profile. It also depends on how key functional modules, including signal transduction, protein homeostasis, metabolic stress responses, and epigenetic reprogramming, are regulated by PTMs 25. For example, phosphorylation mainly shapes T-cell receptor (TCR) signaling and its downstream pathways 26, 27. Ubiquitination, glycosylation, palmitoylation, and UFMylation participate in maintaining the homeostasis of inhibitory receptors and other critical proteins 28-31. O-GlcNAcylation, succinylation, lactylation, and ADP-ribosylation can convert metabolic stressors such as nutrient deprivation and lactate accumulation into stable exhaustion programs 32-35. Methylation, acetylation, and SUMOylation promote the stabilization of exhaustion-associated epigenetic states by reshaping chromatin organization and transcriptional control 36-38. PTMs should therefore not be viewed as accessory layers during the development of CD8⁺ Tex. Rather, they are decisive regulatory strata that determine fate commitment and terminal stabilization. A systematic framework for these hierarchical mechanisms is essential for understanding how CD8⁺ T-cell exhaustion is initiated, maintained, and stabilized.

## 3. Layered PTM mechanisms driving CD8⁺ T-cell exhaustion

**Figure 2:**
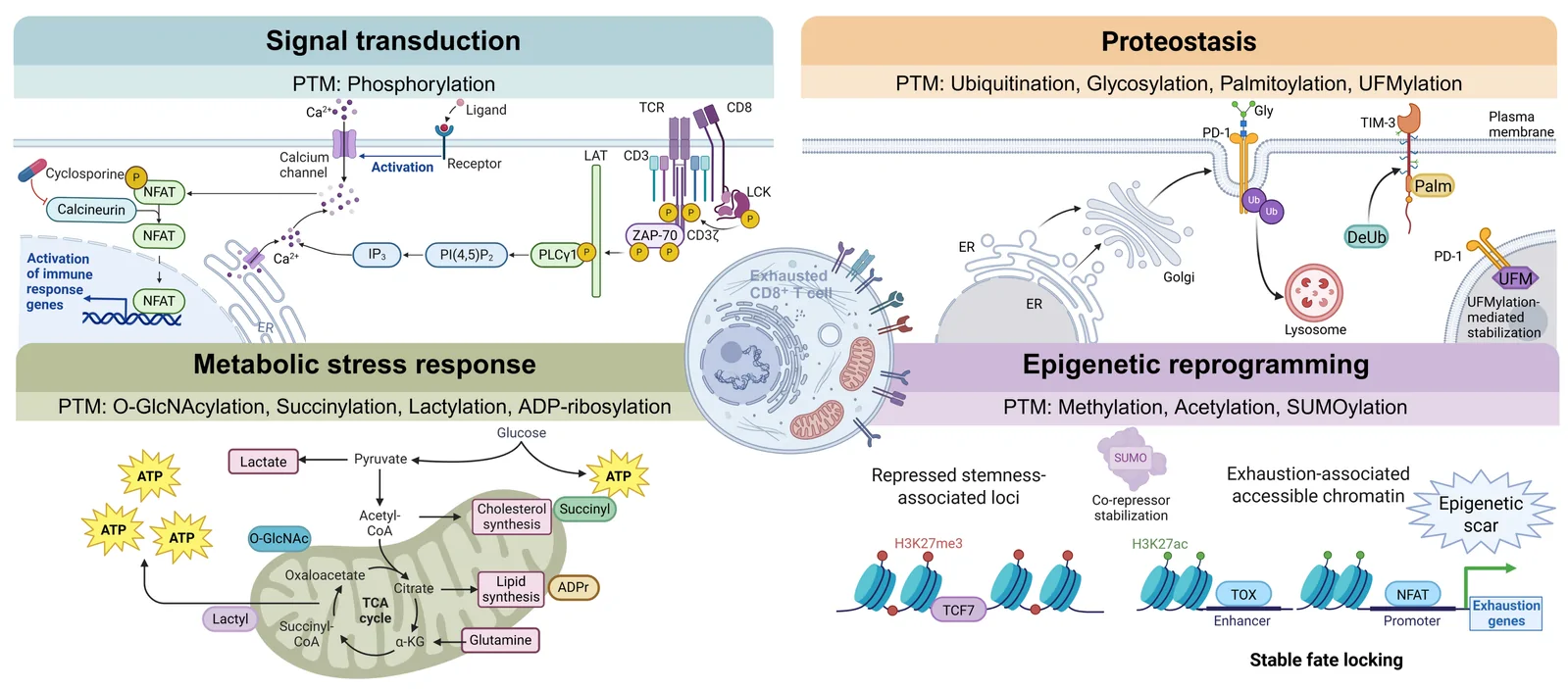
Four functional PTM layers that drive CD8⁺ T-cell exhaustion. Phosphorylation resets activation thresholds through signal transduction. Ubiquitination, glycosylation, palmitoylation, and UFMylation regulate the homeostasis of key proteins. O-GlcNAcylation, succinylation, lactylation, and ADP-ribosylation convert metabolic stress into stable exhaustion programs. Methylation, acetylation, and SUMOylation promote the formation of epigenetic scars. These PTM layers act cooperatively to drive progressive fixation of terminal exhaustion in CD8⁺ T cells.

PTMs are covalent chemical modifications that are introduced at specific amino acid residues after protein synthesis through enzyme-dependent or enzyme-independent reactions, including the addition of phosphate, acetyl, or ubiquitin groups 7. During the development of CD8⁺ Tex driven by persistent antigenic stimulation and TME pressure, PTMs operate in a layered manner. Based on the post-translational modification categories and their predominant functions described above, this section proceeds sequentially. First, we examine how chronic stimulation is sensed through phosphorylation-mediated signal transduction and how activation thresholds are reset. We then describe how inhibitory input is durably maintained at the level of checkpoint molecules and the homeostasis of key proteins through ubiquitination, glycosylation, palmitoylation, and UFMylation. Next, we address how metabolic stress is translated into stable exhaustion programs through metabolism-sensitive post-translational modifications, including O-GlcNAcylation, succinylation, lactylation, and ADP-ribosylation. Finally, we consider how these changes become further fixed at the epigenetic level through methylation, acetylation, and SUMOylation, leading to a relatively stable terminally exhausted state (**Fig. 2**).

### 3.1 Signal transduction: phosphorylation

**Figure 3:**
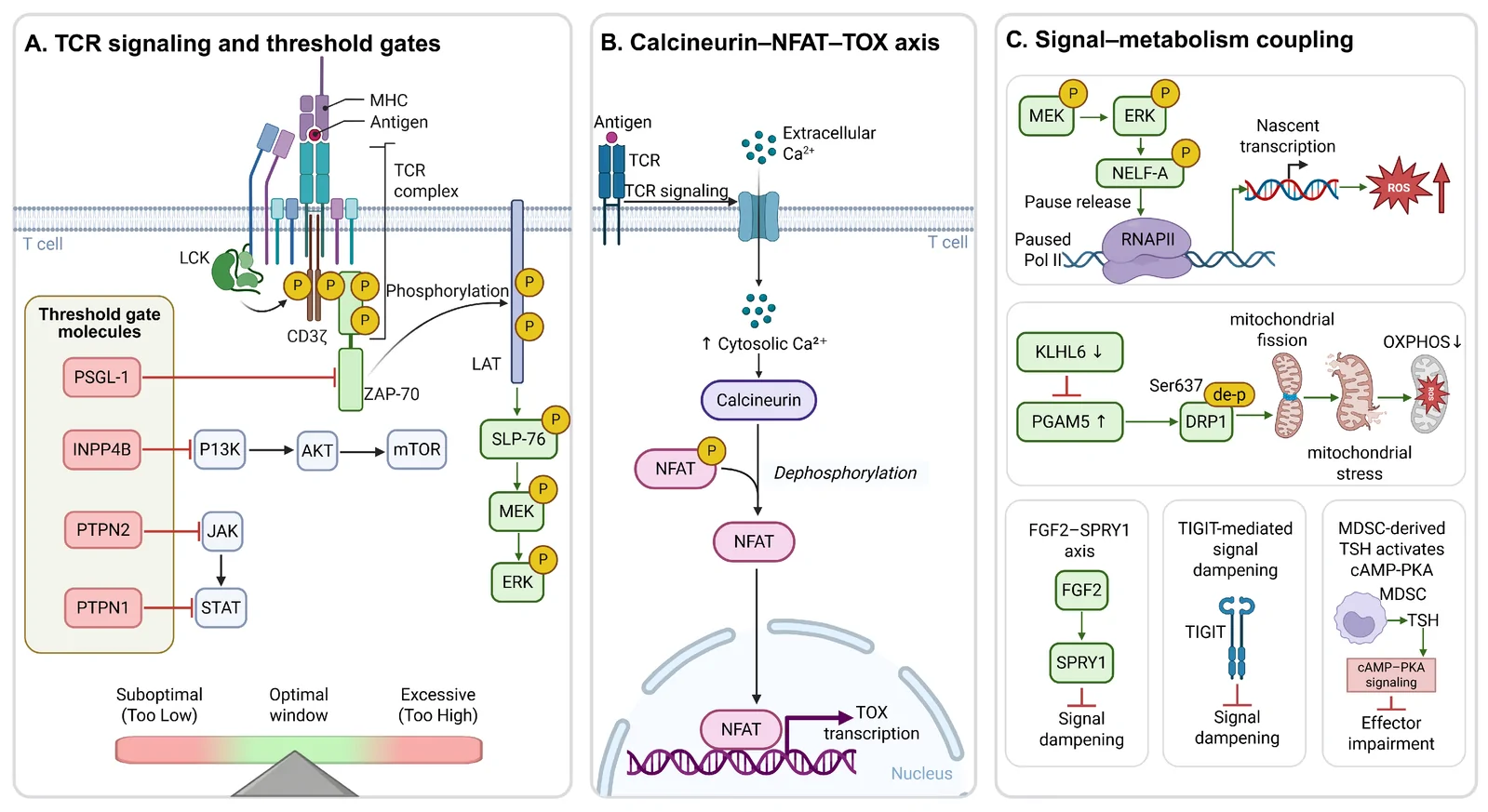
Phosphorylation-mediated signaling control in CD8⁺ T-cell exhaustion. (A) Proximal TCR signaling is transmitted through the LCK-CD3ζ-ZAP-70-LAT-SLP76-MEK-ERK cascade, whereas threshold-gating molecules such as PSGL-1, INPP4B, PTPN2, and PTPN1 constrain PI3K-AKT-mTOR or JAK-STAT branches and reset the activation window. (B) Persistent TCR stimulation induces Ca²⁺ influx and activates calcineurin, which dephosphorylates nuclear factor of activated T cells (NFAT), promotes its nuclear translocation, and drives TOX transcription, thereby coupling chronic stimulation to exhaustion-associated transcriptional programs. (C) Aberrant phosphorylation further links chronic stimulation to transcriptional and metabolic burden. The MEK-ERK-NELF-A axis promotes RNA polymerase II pause release and nascent transcription, together with increased reactive oxygen species (ROS). Upregulation of PGAM5 and dephosphorylation of DRP1 at Ser637 promote mitochondrial fission, mitochondrial stress, and reduced oxidative phosphorylation (OXPHOS). Signals originating from fibroblast growth factor 2 (FGF2)-SPRY1, TIGIT, and myeloid-derived suppressor cell (MDSC)-derived TSH/cAMP-PKA pathways further suppress phosphorylation output and weaken effector function.

Phosphorylation is the covalent attachment of a phosphate group from adenosine triphosphate (ATP) to serine (Ser), threonine (Thr), or tyrosine (Tyr) residues of substrate proteins by protein kinases, whereas protein phosphatases catalyze dephosphorylation at the corresponding sites 39. Together, these opposing reactions create a high-turnover writing-and-erasing cycle that makes phosphorylation the most immediate and sensitive regulatory mode within T-cell signaling networks 40. Under persistent antigenic stimulation, phosphorylation resets the intensity and duration of proximal TCR signals and their downstream pathways, thereby converting recurrent exogenous input into cumulative functional deviation (**Fig. 3**).

Persistent stimulation can therefore be converted into transcriptional burden and metabolic stress. Sustained mitogen-activated protein kinase kinase (MEK) activation increases phosphorylation of the Ser5 and Ser2 residues within the carboxy-terminal domain of RNA polymerase II (RNAPII-CTD), thereby driving energy-intensive nascent transcription and protein synthesis, together with reactive oxygen species accumulation and metabolic impairment, and ultimately biasing CD8⁺ T cells toward terminal exhaustion 26. Downregulation or loss of kelch-like family member 6 (KLHL6) also induces dephosphorylation of dynamin-related protein 1 (DRP1) at Ser637, which promotes excessive mitochondrial fission, loss of membrane potential, and decreased oxidative phosphorylation, thereby driving deeper exhaustion in tumor-infiltrating CD8⁺ T cells 41. Phosphorylation therefore determines not only whether signaling occurs, but also whether persistent stimulation can be converted into a transcriptional and metabolic burden that the cell can no longer tolerate.

At the receptor-proximal level, phosphorylation imbalance is reflected in resetting of the T-cell activation window 42. Sustained Ca²⁺ influx drives dephosphorylation and nuclear translocation of NFAT, which then induces TOX, TOX2, and PD-1 expression, thereby forming a critical bridge between chronic stimulation and exhaustion-associated transcriptional programs 43. In parallel, P-selectin glycoprotein ligand 1 (PSGL-1) suppresses proximal phosphorylation cascades involving phosphorylated ζ-chain-associated protein kinase 70 (p-ZAP70), extracellular signal-regulated kinase 1/2 (ERK1/2), and protein kinase B (AKT), thereby restricting signal amplification and stabilizing the inhibitory receptor phenotype 44. Phosphorylation of Y-box-binding protein 1 (YBX1) at Ser102 by AKT1 activates hypoxia-inducible factor-2α (HIF-2α) and promotes CD8⁺ Tex 45. Folic acid has also been reported to enhance AKT phosphorylation and activate Notch receptor 1 (NOTCH1) signaling, thereby facilitating CD8⁺ Tex formation 46. Distinct from this downstream AKT-associated pro-exhaustion mechanism, the intracellular domain of basigin (CD147) acts at the level of proximal TCR signaling by interacting with CD3ε, CD3ζ, and lymphocyte-specific protein tyrosine kinase (LCK), enhancing CD3ζ/LCK/ZAP70 phosphorylation and promoting LCK/ZAP70 recruitment to CD3ζ, thereby causing proximal signaling overload and accumulation of tumor-infiltrating CD8⁺ Tex 47. These findings suggest that commitment toward CD8⁺ Tex is determined not by the absolute strength of proximal TCR output alone, but by whether phosphorylation output remains within a range that sustains effector function without causing chronic overload.

This threshold shift is not limited to the proximal TCR machinery. It is further reinforced across broader signaling networks by the concerted action of intrinsic negative regulators and extrinsic suppressive signals. Inositol polyphosphate 4-phosphatase type II B (INPP4B) dephosphorylates phosphatidylinositol (3,4)-bisphosphate (PI(3,4)P_2_) into phosphatidylinositol 3-phosphate (PI(3)P), weakens TCR/CD28-induced PI3K-AKT activation, and positively correlates with exhaustion scores and *PDCD1* expression in single-cell analyses 48. Protein tyrosine phosphatase non-receptor type 2 (PTPN2) and PTPN1, also known as PTP1B, increase the activation threshold of CD8⁺ T cells and consolidate exhausted phenotypes by dephosphorylating proximal TCR kinases and inputs to Janus kinase (JAK)-signal transducer and activator of transcription (STAT) signaling 49. Stromal fibroblast growth factor 2 (FGF2) promotes dephosphorylation of linker for activation of T cells (LAT) and SH2-domain-containing leukocyte protein of 76 kDa (SLP-76) through a CBL-mediated negative-feedback loop, while simultaneously increasing inhibitory phosphorylation of LCK at Tyr505, thereby driving terminal exhaustion 27. Signaling related to the ITIM-containing receptor TIGIT further reinforces this suppressed state by weakening TCR-induced ERK phosphorylation and reducing IFN-γ production 50. Phosphorylation-dependent threshold resetting is therefore not a transient event. It evolves into a durable hyporesponsive state through the joint action of intrinsic brakes and microenvironment-derived inhibitory inputs.

Phosphorylation networks also extend into intercellular suppressive signaling within the TME. Tumor-associated signals activate transforming growth factor-β (TGF-β)-SMAD2/3 and ERK1/2 phosphorylation cascades in Schwann cells, which in turn induce upregulation of *PDCD1* and *NT5E* in CD8⁺ T cells. *NT5E* encodes ecto-5′-nucleotidase CD73, which cooperates with CD39 to convert extracellular ATP-derived AMP into immunosuppressive adenosine, thereby promoting CD8⁺ Tex 51. Myeloid-derived suppressor cells (MDSCs) secrete thyroid-stimulating hormone (TSH) and cooperate with tumor-derived exosomes to induce phosphorylated cAMP response element-binding protein (p-CREB), thereby transcriptionally activating genes encoding co-inhibitory receptors such as *PDCD1* and *HAVCR2* 52. IFN-γ can also promote phosphorylation of sphingosine kinase 1 (SPHK1) at Ser225 in tumor-associated macrophages (TAMs), amplifying myeloid-cell recruitment and accelerating functional exhaustion of CD8⁺ tumor-infiltrating lymphocytes (TILs) 53. C-C motif chemokine ligand 23 (CCL23) secreted by M2-like TAMs activates the CCR1-glycogen synthase kinase 3β (GSK-3β) phosphorylation axis and is accompanied by coordinated upregulation of multiple checkpoint molecules 54. A Siglec-9⁺ TAM-associated SHP-1 pathway has likewise been closely linked to the development of CD8⁺ Tex 55. These observations indicate that phosphorylation converts stromal and myeloid cells into sustained amplifiers of inhibitory signaling, thereby further shifting the activation window from outside the T cell.

The supply of inhibitory ligands from tumor cells can also be persistently reinforced through phosphorylation, which further stabilizes the already established hyporesponsive state 56. In tumor cells, PTPN22 blocks K48-linked ubiquitin-mediated degradation of PD-L1 by promoting dephosphorylation of CBL at Tyr700, thereby stabilizing PD-L1 expression 57. Under high-glucose conditions, ERK2 phosphorylates V-domain Ig suppressor of T-cell activation (VISTA) at Ser248, recruits the deubiquitinase USP22, and stabilizes VISTA through deubiquitination, which promotes CD8⁺ Tex 58. ERK1/2 also phosphorylates CREB1 at Ser133, upregulates USP8, reduces ubiquitination of TGF-β receptor 2 (TGFBR2), and increases its stability, thereby inducing an exhausted phenotype 59. Phosphorylation of basic leucine zipper ATF-like transcription factor 2 (BATF2) at Ser227 is a key event for promoting stimulator of interferon genes (STING) oligomerization and amplifying type I interferon (IFN-I) responses. Consistently, high-glutamine states induced by tumor cells suppress BATF2 expression through epigenetic mechanisms, thereby weakening effector expansion and promoting exhaustion 60. Increased tumor-cell levels of phosphorylated SHP2 at Tyr542 are also associated with upregulation of exhaustion markers such as *HAVCR2* and *PDCD1* in CD8⁺ Tex 61. Loss of tumor-cell CD58 results in excessive p-STAT1 activation and concomitant PD-L1 upregulation, thereby strengthening the immunosuppressive barrier 62. Immune-derived signals can, in turn, further shape the inhibitory output of tumor cells. Extracellular granzyme K (GZMK) triggers AKT-GSK3β-β-catenin and JAK2-STAT1 phosphorylation cascades, upregulates tumor-cell PD-L1, and promotes CD8⁺ Tex formation 63. Non-canonical phosphorylation of STAT1 at Ser727 can likewise promote CD8⁺ Tex by upregulating CD276 64. Similar roles have been reported for phosphorylation-related pathways involving STAT3, GSK-3β, β-catenin, AKT, NF-κB, immunoreceptor tyrosine-based inhibitory motifs (ITIMs), and protein phosphatase 1A (PP1A), all of which participate in PD-L1 upregulation in tumor cells and reinforcement of an inhibitory TME. Phosphorylation therefore promotes CD8⁺ Tex not only through T-cell-intrinsic signaling but also by sustaining the supply of inhibitory ligands from tumor cells.

Phosphorylation-related suppressive signals can even spread beyond the tumor through long-range intercellular transfer. Tumor-derived extracellular vesicles carrying phosphorylated interferon-γ receptor 1 (IFNGR1) can trigger a JAK1-STAT1 phosphorylation cascade, upregulate PD-L1, and induce an exhaustion program in CD8⁺ T cells 65. At the same time, acidic pH and interleukin-8 (IL-8) within the microenvironment enhance STAT1/STAT3 phosphorylation and are associated with upregulation of the PD-L1/PD-1 axis 66. These results indicate that phosphorylation-mediated inhibitory remodeling is not confined to the local tumor niche. It can also precondition distant sites and create molecular conditions that favor exhaustion before T cells arrive, thereby extending the impact of persistent antigenic stimulation beyond local spatial and temporal boundaries.

Taken together, the central role of phosphorylation in CD8⁺ Tex lies not in a simple increase or decrease in the activity of any single pathway, but in the continuous reshaping of T-cell responses to chronic stimulation. Conversion of persistent stimulation into transcriptional and metabolic burden, deviation of the proximal TCR activation window, cooperative reinforcement by intrinsic and extrinsic inhibitory signals, and intercellular amplification within both local and distant niches together form the upstream mechanism that initiates the exhaustion program.

### 3.2 Protein homeostasis

**Figure 4:**
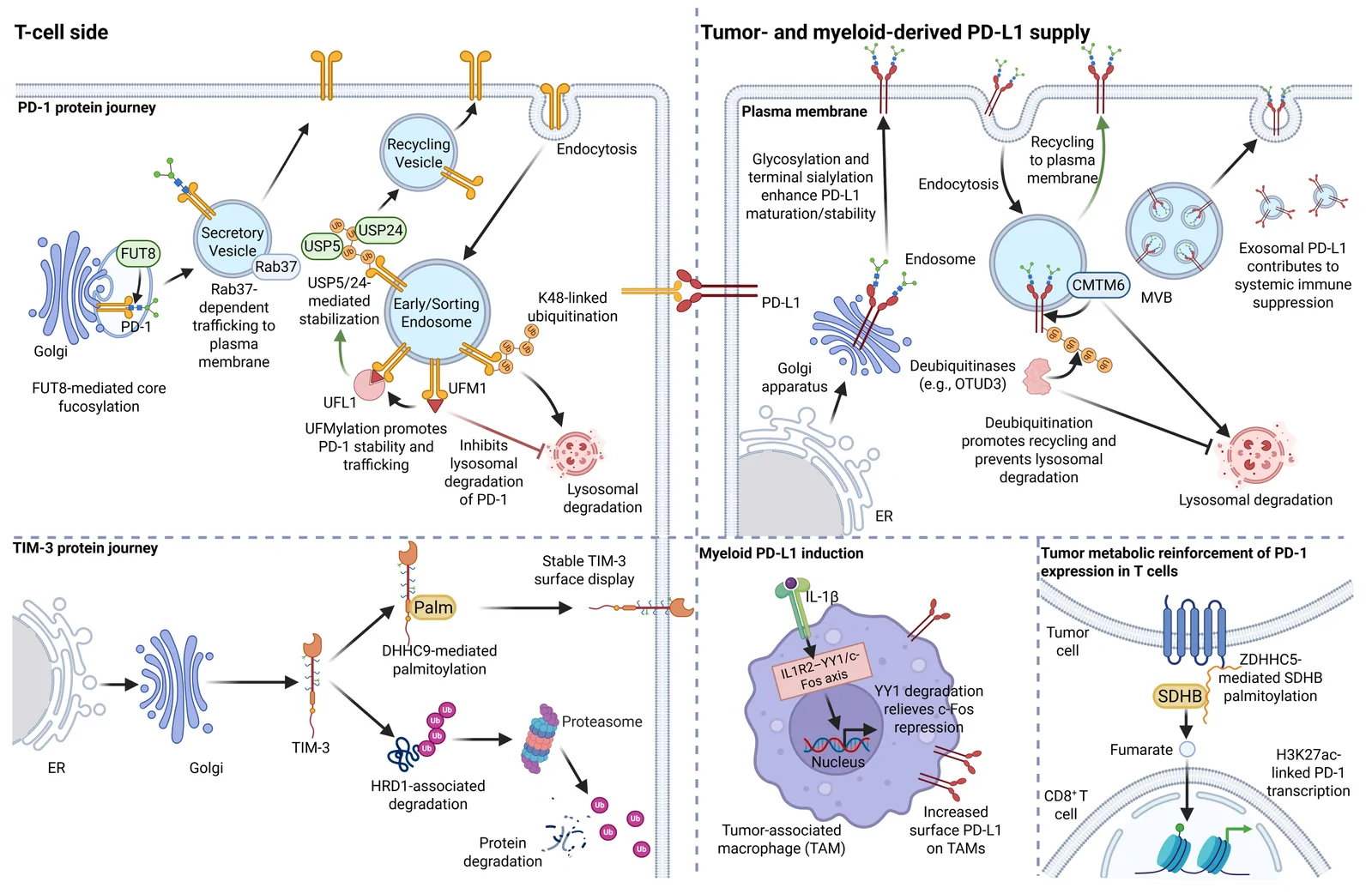
Sustained preservation of inhibitory input in CD8⁺ T-cell exhaustion through ubiquitination, glycosylation, palmitoylation, and UFMylation. Upper left: in T cells, PD-1 undergoes FUT8-mediated core fucosylation, Rab37-dependent membrane transport, and USP5/USP24-mediated recycling and stabilization. UFL1-UFM1-mediated UFMylation further promotes PD-1 stability and trafficking while suppressing lysosomal degradation, whereas K48-linked ubiquitination promotes degradation. Upper right: Tumor- and myeloid cell-derived PD-L1 is stabilized and matured by glycosylation and terminal sialylation. CMTM6 and deubiquitination facilitate recycling after endocytosis and prevent lysosomal degradation, whereas exosomal PD-L1 further strengthens systemic immune suppression. Lower left: TIM-3 remains stably expressed on the membrane after DHHC9-mediated palmitoylation, whereas HRD1-associated ubiquitination promotes its proteasomal degradation. Lower center: in TAMs, the IL-1β-IL1R2-YY1-c-Fos axis promotes surface PD-L1 upregulation. Lower right: tumor-derived metabolic input can further enhance PD-1 expression in T cells through ZDHHC5-mediated palmitoylation of SDHB, fumarate accumulation, and H3K27ac-associated activation of *PDCD1* transcription.

Suppressive signal transduction alone cannot fully explain why the exhausted phenotype is maintained over long periods. Transient inhibitory input becomes chronic pressure because the processing, maturation, membrane localization, stability, and degradation of inhibitory receptors such as PD-1 and TIM-3 and their ligands, including PD-L1, are continuously regulated 67. In addition, critical proteins functionally coupled to these pathways must also be maintained at high steady-state levels to sustain inhibitory input. At this layer, ubiquitination, glycosylation, palmitoylation, and UFMylation jointly shape the homeostasis of checkpoints and associated proteins through control of degradation, biosynthetic processing, membrane presentation, and PTM crosstalk (**Fig. 4**), thereby preserving suppressive input over time.

#### 3.2.1 Ubiquitination

Ubiquitination is mediated by the E1-E2-E3 enzyme cascade, which covalently conjugates ubiquitin to lysine residues on substrate proteins. Depending on chain type and context, it can direct proteasomal degradation, receptor turnover, or reorganization of signaling complexes, whereas deubiquitinases (DUBs) reversibly remove ubiquitin chains. Ubiquitination is therefore a key determinant of protein half-life and cell-surface receptor homeostasis 68. In CD8⁺ Tex, its major significance lies in stabilizing checkpoint molecules and other critical proteins, thereby preserving inhibitory input and progressively fixing a stable functionally suppressed state.

At the T-cell-intrinsic level, inflammatory signals within the TME can alter PD-1 protein homeostasis. CAF-derived IL-8 downregulates the E3 ligase FBXO38 in CD8⁺ T cells and suppresses ubiquitin-mediated degradation of PD-1 25, 69. Interleukin-6 (IL-6) derived from TAMs and tumor cells induces the deubiquitinase USP24 through STAT3/NF-κB signaling, enhances removal of K48-linked chains from PD-1, and markedly prolongs the PD-1 half-life, thereby sustaining a PD-1^hi^ exhausted TIL state 70. In addition, ERK-mediated site-specific phosphorylation promotes the interaction between PD-1 and the deubiquitinase USP5, leading to removal of degradation-associated ubiquitin chains and further stabilization of the PD-1 protein 28. Defects in core ubiquitin-system components similarly impair PD-1 degradation and allow PD-1 accumulation in TILs 71. Sustained PD-1 expression is thus not only a transcriptional phenomenon, but also a steady-state consequence of restricted degradation and enhanced protein stability.

Persistent suppressive input is also reflected at the ligand-supply side. OTU domain-containing protein 3 (OTUD3) removes K48-linked ubiquitin chains from PD-L1 and blocks its proteasomal degradation 72. Extracellular GZMK activates protease-activated receptor 2 (PAR2) in tumor cells, inhibits PD-L1 ubiquitin-dependent degradation, and maintains high PD-L1 abundance 63. Similar deubiquitinating mechanisms can affect non-classical checkpoints. USP2a removes K48/K63-linked chains from B7 homolog 4 (B7-H4), increases its stability, and is associated with exhaustion-related infiltrates 73. Moreover, sustained expression of inhibitory ligands is not limited to single-cell-intrinsic processes. It can be amplified through cross-cellular cooperation. TAM-derived IL-1β activates IL-1R2, whose intracellular domain drives ubiquitin-proteasome-mediated degradation of YY1, thereby synchronously increasing PD-L1 in TAMs and tumor cells and deepening TIL exhaustion 74.

Once inhibitory ligand supply is persistently maintained, changes in the homeostasis of T-cell-intrinsic signaling molecules and exhaustion-associated proteins further determine whether the exhaustion program can be sustained. Upregulation of the E3 ligase Pellino 1 (Peli1) promotes K48-linked ubiquitination and degradation of protein kinase C-θ (PKC-θ), thereby weakening TCR signaling and maintaining the PD-1^hi^ Tex phenotype 75. By contrast, loss of the E3 ligases NEURL3, RNF149, and WSB1 causes abnormal K48-linked ubiquitination, accumulation of unfolded proteins, and enhanced unfolded protein response (UPR), leading to loss of the Tpex pool and accelerated terminal exhaustion 76. The deubiquitinase USP7 stabilizes recombination signal binding protein for immunoglobulin kappa J region (RBPJ) through deubiquitination and drives an interferon regulatory factor 4 (IRF4)/tumor necrosis factor receptor superfamily member 1B (TNFRSF1B) axis that maintains exhaustion programs 77. In parallel, there are also ubiquitin-dependent restraints that limit terminal exhaustion. KLHL6 promotes K48-linked ubiquitination and degradation of TOX, thereby constraining the transition from Tpex to Tex^term^ 41.

Notably, ubiquitin-dependent control extends beyond classical signaling or transcriptional regulators to metabolic and mitochondrial nodes that determine whether suppression can be maintained. Tumor-derived exosomes deliver β-transducin repeat-containing protein (β-TrCP) into CD8⁺ T cells, which induces ubiquitination-mediated degradation of yes-associated protein 1 (YAP1), suppresses expression of amino acid transporters SLC7A5 and SLC38A1, and is accompanied by increased inhibitory receptor expression and impaired effector function 78. Valosin-containing protein (VCP) stabilizes glycerol-3-phosphate dehydrogenase 1-like protein (GPD1L) by preventing its ubiquitin-dependent degradation, leading to glycerol-3-phosphate accumulation and enhanced inhibitory phosphorylation of LCK at Tyr505. In this way, abnormal metabolic protein homeostasis is converted into sustained hyporesponsive signaling 79. NFATc1 upregulates the mitochondrial deubiquitinase USP30, which counteracts PINK1/Parkin-mediated ubiquitination of mitochondrial proteins, inhibits mitophagy, promotes accumulation of damaged mitochondria, and thereby drives both formation and maintenance of CD8⁺ Tex 80. Similarly, dipeptidyl peptidase 7 (DPP7) in TAMs blocks TRIM25-mediated ubiquitination of carnitine palmitoyltransferase 1A (CPT1A), stabilizes CPT1A, enhances fatty acid β-oxidation (FAO)/OXPHOS, and maintains an immunosuppressive macrophage phenotype that favors accumulation of CD8⁺ Tex 81. K48-linked ubiquitination and proteasomal degradation of tropomyosin receptor kinase A (TRKA) suppress glycolysis in CD8⁺ T cells and promote exhaustion 82. Ubiquitination therefore sustains inhibitory input not only by stabilizing checkpoints themselves, but also by fixing a broad set of proteins that regulate TCR output, mitochondrial quality control, and immunosuppressive myeloid states.

Tumor-driven abnormal signaling can likewise induce ubiquitination imbalance in critical proteins and thereby exacerbate CD8⁺ Tex. High expression of the long intergenic non-coding RNA LINC01116 in tumor cells blocks RAD18-mediated ubiquitination and degradation of EWS RNA-binding protein 1 (EWSR1). This promotes the preferential uptake of long-chain fatty acids, especially linoleic acid, by tumor cells, causing lipid nutrient competition with CD8⁺ T cells in the TME and ultimately exacerbating CD8⁺ Tex 83. Circulating tumor cells induce CD8⁺ Tex by triggering CBL-mediated ubiquitination and degradation of epidermal growth factor receptor (EGFR) 84. In tumor cells, follistatin-like 3 (FSTL3) translocates into the nucleus, binds c-Myc, prevents its ubiquitin-mediated degradation, upregulates PD-L1, and weakens responsiveness to anti-PD-1 therapy 85. The tumor metabolite kynurenine suppresses aryl hydrocarbon receptor (AhR) ubiquitin-dependent degradation in CD8⁺ T cells and promotes its nuclear translocation, leading to increased PD-1, LAG-3, and TIM-3 and reduced IFN-γ/TNF production 86. In tumor cells, fucosyltransferase 2 (FUT2) promotes FBXO2-mediated ubiquitination and degradation of nuclear receptor subfamily 2 group F member 2 (NR2F2). Radiotherapy suppresses FUT2 through METTL14-dependent N6-methyladenosine (m6A), ultimately inducing CD8⁺ Tex 87. High expression of the E3 ligase bifunctional apoptosis regulator (BFAR) in tumor cells drives K48-linked ubiquitination and degradation of pre-mRNA processing factor 19 (PRPF19), promoting surface PD-L1 expression on tumor-associated neutrophils 88. Likewise, high tumor-cell expression of ependymin-related protein 1 (EPDR1) interferes with TRIM21-mediated ubiquitination and autophagic degradation of IKKβ, enhances NF-κB-dependent PD-L1 transcription, and promotes CD8⁺ Tex 89. Ring finger protein 26 (RNF26) also promotes K48-linked ubiquitin-mediated degradation of glucose-regulated protein 78 (GRP78), induces persistent endoplasmic reticulum stress, upregulates PD-L1, and deepens CD8⁺ Tex 90. These observations indicate that tumor-cell ubiquitin abnormalities do not simply affect scattered metabolic or stress pathways. They stabilize key proteins that control checkpoint expression, antigen presentation, and recruitment of immunosuppressive cells, thereby maintaining suppressive input at high load.

Taken together, the central role of ubiquitination in CD8⁺ Tex is to convert transient inhibitory input into a sustained molecular state by controlling the degradation and stability of PD-1, PD-L1, and other critical proteins.

#### 3.2.2 Glycosylation

Whereas ubiquitination mainly determines degradation and retention of checkpoints and related proteins, glycosylation primarily controls their maturation and cell-surface homeostasis along the endoplasmic reticulum-Golgi route. N-glycosylation, O-glycosylation, and related glycan modifications participate in protein folding, quality control, intracellular trafficking, membrane localization, and ligand recognition, and thus directly determine the maturation efficiency and surface availability of membrane proteins 91. In CD8⁺ Tex, glycosylation is critical because it maintains the processing, trafficking, and membrane residence of checkpoint molecules and other key proteins, thereby supporting persistent inhibitory input and stabilizing the exhausted phenotype.

At the T-cell-intrinsic level, abnormal glycosylation can sustain inhibitory receptor burden by affecting both effector-molecule secretion and checkpoint membrane homeostasis. In tumor-infiltrating CD8⁺ T cells, downregulation of the oligosaccharyltransferase (OST) complex leads to insufficient occupancy of N-glycosylation sites and creates a mismatch in which IFN-γ transcripts increase but protein secretion declines. Because IFN-γ exerts negative-feedback inhibition on *PDCD1*, this secretory defect weakens PD-1 restraint and promotes exhaustion 92. Glycosylation can also directly determine the stable membrane residence of PD-1. FUT8-mediated core fucosylation maintains PD-1 surface expression, whereas FUT8 inhibition reduces membrane PD-1 and enhances T-cell activation 93. Complementing this glycan effect, PD-1 glycosylation facilitates its loading into Rab37-positive vesicles and subsequent plasma membrane presentation. Consistently, Rab37 deficiency reduces PD-1 and TIM-3 expression and is accompanied by restoration of T-cell effector function 29. In addition, glycosylation may interact with other PTMs to influence the steady-state levels of exhaustion-associated proteins. Short-term fasting blocks N-glycosylation of the fatty acid transporter CD36, increases AMP-activated protein kinase (AMPK) phosphorylation, reduces UFM1 modification of the deubiquitinase USP7, enhances ubiquitin-mediated USP7 degradation, and ultimately promotes degradation of RBPJ and suppression of the IRF4/TNFRSF1B exhaustion axis 77. Thus, glycosylation prolongs suppressive signaling both by maintaining maturation and membrane presentation of receptors such as PD-1 and by influencing key homeostatic proteins through PTM crosstalk.

Persistent suppressive input is also reflected on the supply side through stable maintenance of inhibitory ligands and related membrane proteins. In tumor cells, either the β-glucuronidase (GUSB) H351Q mutation or glycosyltransferase 8 domain-containing protein 2 (GLT8D2) promotes aberrant N-glycosylation of PD-L1, thereby enhancing PD-L1 stability and increasing infiltration by exhausted CD8⁺ T cells 94. Loss of tuberous sclerosis complex subunit 1 (TSC1) enhances α2,6-sialylation of PD-L1, increases its affinity for PD-1, and ultimately promotes CD8⁺ Tex formation 95. Beyond PD-L1, aberrant glycosylation can also sustain suppressive input by stabilizing other immunoregulatory membrane proteins at the cell surface. FUT8-mediated core fucosylation of semaphorin 7A (SEMA7A) in tumor cells promotes differentiation of CD8⁺ T cells along the exhaustion trajectory and increases the expression of multiple checkpoint molecules 96. These data indicate that glycosylation abnormalities in tumor cells do not simply increase the abundance of a single ligand. Rather, they maintain long-term suppressive load by enhancing the maturation, surface stability, and receptor-binding capacity of inhibitory molecules.

Notably, the glycome of intratumoral CD8⁺ T cells changes early during exhaustion. Increased β1,6-GlcNAc-branched N-glycans are associated with a PD-1⁺TIM-3⁺ phenotype 97. This finding suggests that glycosylation not only maintains already established checkpoint homeostasis, but may also preconfigure a cell state that is intrinsically more prone to acquiring a persistent inhibitory receptor phenotype.

Taken together, the key role of glycosylation in CD8⁺ Tex is to extend suppressive input over time and stabilize it in space by controlling the maturation, intracellular trafficking, membrane presentation, and receptor-ligand binding capacity of checkpoints and associated proteins.

#### 3.2.3 Palmitoylation

Palmitoylation is a reversible lipid modification in which palmitate and related long-chain fatty acids are covalently linked to cysteine residues through thioester bonds. This modification amplifies signaling output by altering membrane affinity, subcellular localization, and protein stability, thereby reinforcing persistent inhibitory effects mediated by checkpoints and associated proteins 98. Palmitoylation is mainly written by the zinc finger DHHC-type palmitoyltransferase family (ZDHHCs) and erased by depalmitoylating enzymes, forming a high-turnover cycle 99.

The contribution of this PTM to CD8⁺ Tex first becomes evident at the level of inhibitory receptors themselves. In TIM-3-expressing CD8⁺ T cells, the palmitoyltransferase DHHC9 has been shown to catalyze the palmitoylation of TIM-3 at Cys296, thereby suppressing its ubiquitination-mediated degradation and maintaining TIM-3 stability and surface expression, ultimately contributing to CD8⁺ Tex 30. Beyond directly stabilizing checkpoint receptors, palmitoylation can indirectly sustain suppressive programs by stabilizing key metabolic signaling nodes. ZDHHC5-mediated palmitoylation of succinate dehydrogenase complex subunit B (SDHB) prevents its lysosomal degradation, promotes fumarate production, enhances H3K27ac-dependent PD-1 transcription, and ultimately facilitates CD8⁺ Tex formation 100. Likewise, after palmitate enters CD8⁺ T cells through CD36, it increases STAT3 palmitoylation and membrane localization, transcriptionally activates TOX, and promotes differentiation toward Tex^term^ 101. These findings indicate that palmitoylation not only extends the membrane half-life of inhibitory receptors, but also stabilizes upstream proteins that continuously support checkpoint expression and exhaustion-associated transcriptional programs.

Taken together, the central role of palmitoylation in CD8⁺ Tex is to prolong the effective lifetime of inhibitory molecules and exhaustion-associated nodes by enhancing membrane residence, suppressing degradation, and stabilizing functional protein sites.

#### 3.2.4 UFMylation

UFMylation is a ubiquitin-like modification mediated by UFM1. In the present context, it appears to reinforce protein homeostasis mainly through crosstalk with other PTMs. Although the available evidence remains limited, its position in CD8⁺ Tex is becoming clearer. UFMylation does not seem to create an entirely independent exhaustion-driving pathway. Rather, it acts as a cross-regulatory component within the homeostatic network that controls checkpoints and related proteins 102. By directly stabilizing PD-1 and acting in concert with glycosylation and ubiquitination, UFMylation helps preserve suppressive molecules and prolong the period during which inhibitory input is stably maintained 31, 67.

Taken together, ubiquitination, glycosylation, palmitoylation, and UFMylation act through distinct molecular mechanisms, yet they converge on a common outcome in CD8⁺ Tex: maintenance of the homeostasis of checkpoint molecules and associated critical proteins. At this layer, the threshold shift initiated by altered signaling is translated into durable checkpoint overexpression and abnormal steady-state control of key proteins, thereby forming a major basis for the maintenance of CD8⁺ Tex.

### 3.3 Metabolic stress responses

**Figure 5:**
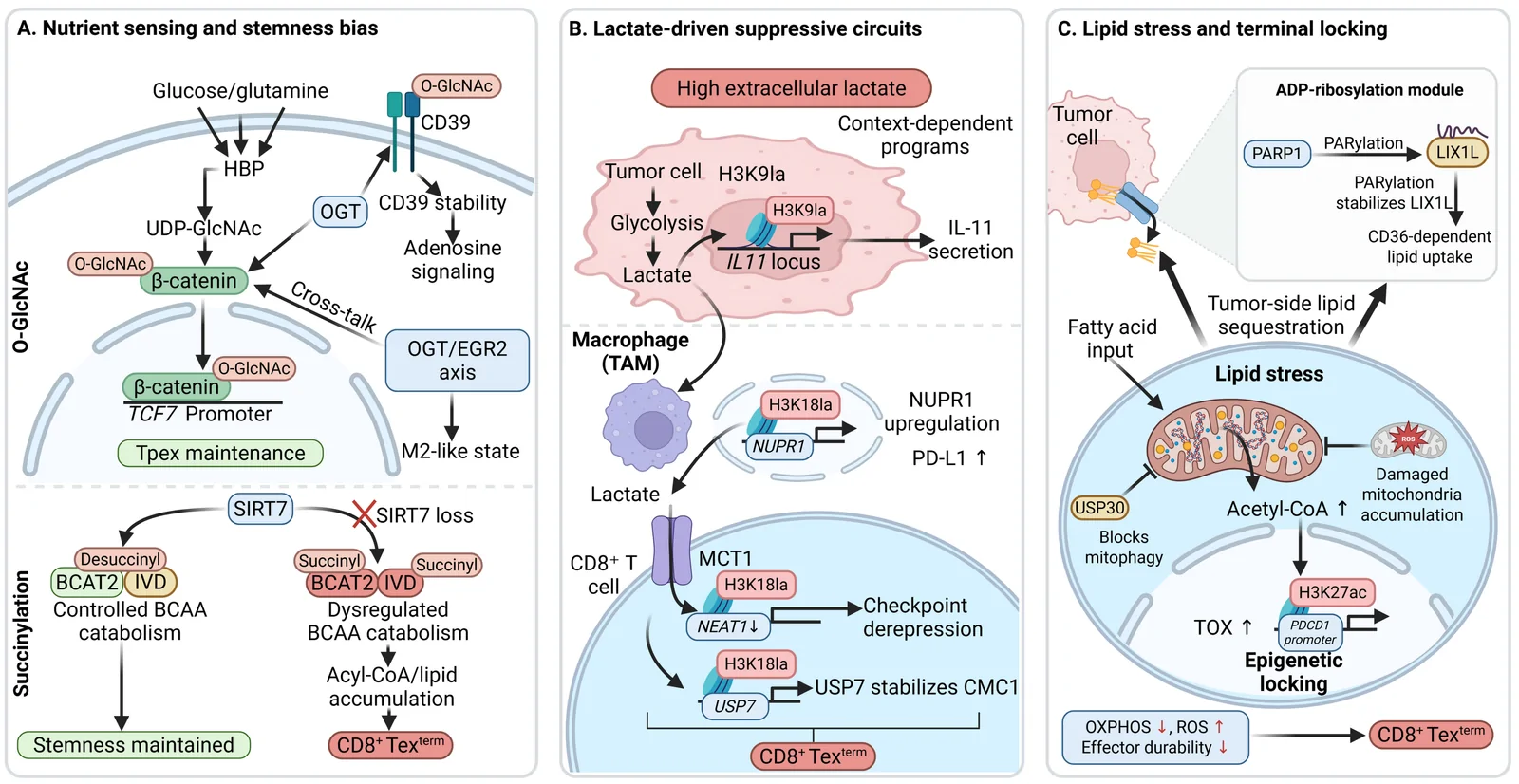
O-GlcNAcylation, succinylation, lactylation, and ADP-ribosylation mediate metabolic stress responses in CD8⁺ T-cell exhaustion. (A) O-GlcNAcylation and succinylation inscribe nutrient sensing into stemness maintenance and immunosuppressive bias. The hexosamine biosynthetic pathway (HBP)-O-GlcNAc transferase (OGT) axis supports β-catenin/*TCF7*-driven Tpex maintenance and stabilizes CD39 to enhance adenosine signaling. β-Catenin also crosstalks with the OGT-EGR2 axis to promote an M2-like state. Loss of SIRT7 is associated with abnormal succinylation of BCAT2 and IVD, disrupted branched-chain amino acid catabolism, and terminal exhaustion. (B) A high-lactate microenvironment establishes a suppressive circuit among tumor cells, TAMs, and CD8⁺ T cells through lactylation. In tumor cells, H3K9la activates the *IL11* locus and promotes IL-11 secretion. In TAMs, H3K18la-associated *NUPR1* upregulation enhances PD-L1 expression. Lactate uptake through monocarboxylate transporter 1 (MCT1) induces H3K18la-associated *NEAT1* downregulation and *USP7* upregulation in T cells; *USP7* stabilizes CMC1, and these changes promote terminal exhaustion. (C) ADP-ribosylation and lipid stress jointly aggravate mitochondrial imbalance and facilitate terminal locking. Poly(ADP-ribose) polymerase 1 (PARP1)-dependent PARylation stabilizes LIX1L and enhances CD36-dependent lipid uptake. USP30 suppresses mitophagy, leading to accumulation of damaged mitochondria, increased acetyl-CoA, TOX upregulation, and enhanced H3K27ac at the *PDCD1* promoter, ultimately causing reduced OXPHOS, elevated ROS, and formation of CD8⁺ Tex^term^.

The mechanisms operating at the levels of signal transduction and protein homeostasis are not sufficient to explain how metabolic stress is further integrated into a durable exhaustion program. Hypoxia, nutrient restriction, lactate accumulation, lipid overload, and NAD⁺-related stress in the TME do not spontaneously become stable exhausted states. They must first be sensed and inscribed through specific PTMs before they can be deposited as functional defects and stable fate changes. Based on current evidence, O-GlcNAcylation, succinylation, lactylation, and ADP-ribosylation translate metabolic stress into persistent exhaustion programs by reshaping T-cell fate, rewiring metabolic pathways, reinforcing suppressive niches, and disrupting mitochondrial homeostasis, thereby jointly promoting the formation and progression of CD8⁺ Tex (**Fig. 5**).

#### 3.3.1 O-GlcNAcylation

O-GlcNAcylation mainly occurs on serine or threonine residues of nuclear, cytoplasmic, and mitochondrial proteins. It is written by O-GlcNAc transferase (OGT), which uses uridine diphosphate N-acetylglucosamine (UDP-GlcNAc) as a donor, and erased by O-GlcNAcase (OGA) 103. In the context of CD8⁺ T-cell exhaustion, O-GlcNAcylation translates nutrient sensing into T-cell metabolic programming, fate maintenance, and remodeling of immunosuppressive niches, with its functional outcome determined by the modified substrate, cell type, and tumor metabolic context.

At the T-cell-intrinsic level, O-GlcNAcylation links nutrient status to metabolic rewiring and maintenance of a precursor-like program, thereby influencing the tendency of CD8⁺ T cells to differentiate toward exhaustion under chronic stimulation. D-mannose has been shown to reduce glycolysis and enhance mitochondrial function while increasing OGT-mediated O-GlcNAcylation and stabilization of β-catenin. This preserves *Tcf7* expression and epigenetic stemness, thereby promoting a stem-like program and restricting exhaustion differentiation 32. By contrast, substrate-specific O-GlcNAcylation may also support exhaustion-associated phenotypes. Increased OGT activity promotes O-GlcNAcylation of CD39 at Ser239, inscribing elevated hexosamine biosynthetic pathway activity into high CD39 expression, which is accompanied by reduced cytotoxic molecules and increased exhaustion markers 104.

O-GlcNAcylation also acts outside T cells by converting glucose-metabolic signals into tumor-promoting differentiation programs in myeloid cells. OGT-mediated O-GlcNAcylation of early growth response 2 (EGR2) at Ser299 promotes the formation of protumorigenic TAM subsets and thereby enhances CD8⁺ Tex features 105. In tumor cells, O-GlcNAcylation can further reshape secretory programs that support immunosuppressive niches. OGT-mediated O-GlcNAcylation of Wilms tumor 1-associated protein (WTAP), together with USP7-mediated deubiquitination, stabilizes WTAP, drives myeloid macrophages toward an M2-like program, and promotes CD8⁺ Tex 106. Clinical correlation analyses also suggest that high expression of EGF domain-specific O-linked N-acetylglucosamine transferase (EOGT) is associated with CD8⁺ Tex in tumors, although a direct causal mechanism remains to be established 107.

Taken together, O-GlcNAcylation does not uniformly promote CD8⁺ Tex, but shapes exhaustion-related programs in a substrate-, cell-type-, and tumor metabolic context-dependent manner.

#### 3.3.2 Lysine succinylation

Succinylation is an acyl modification that introduces a succinyl group onto the epsilon-amino group of lysine residues. This changes residue charge and thereby influences protein conformation, enzymatic activity, and interaction networks 108. Current direct evidence connecting succinylation to CD8⁺ Tex centers on sirtuin 7 (SIRT7), which restrains branched-chain amino acid catabolism and subsequent lipid accumulation through desuccinylation. Loss of SIRT7 in T cells promotes metabolic imbalance and drives CD8⁺ T cells toward exhaustion 34.

#### 3.3.3 Lactylation

Lactylation is an acyl modification in which a lactyl group is introduced onto the epsilon-amino group of lysine residues. It can form specific epigenetic marks such as H3K9la and H3K18la and translate increased glycolysis and lactate accumulation into altered transcriptional output 109. In CD8⁺ Tex, lactylation can contribute importantly by establishing a lactate-driven suppressive chain among tumor cells, myeloid cells, and T cells, thereby expanding local metabolic pressure into a durable immunosuppressive network.

In tumor cells, lactylation converts metabolic pressure into altered secretory programs and immunosuppressive output 110. Tumor-cell forkhead box K1 (FOXK1)-driven glycolipid metabolic reprogramming increases lactate production and modulates TOX-induced histone lactylation in CD8⁺ T cells, thereby promoting CD8⁺ Tex formation and immune evasion 33. Intratumoral lactate accumulation enriches H3K9la at the IL-11 transcriptional start region, promotes IL-11 secretion, increases multiple inhibitory receptors on CD8⁺ T cells, and suppresses effector output 111. Non-histone lactylation also contributes to this process. In tumor cells, hepatitis B virus (HBV)-induced lactate production promotes lactylation of spectrin alpha, non-erythrocytic 1 (SPTAN1) at K1952/K1957 in an alanyl-tRNA synthetase 1 (AARS1)-dependent manner, thereby facilitating accumulation of CD8⁺ Tex 112. In myeloid cells, tumor-derived lactate enters TAMs and induces H3K18la, which increases PD-L1/PD-L2 expression and ultimately promotes accumulation of PD-1⁺ CD8⁺ Tex 113. Loss of claudin 7 (CLDN7) can also remodel neutrophil metabolism, increase glycolysis and histone lactylation together with PD-L1 upregulation, and thereby induce CD8⁺ Tex 114.

Within CD8⁺ T cells, lactylation under high-lactate conditions can directly reshape exhaustion-associated transcriptional programs and disturb mitochondrial homeostasis. Lactate entry increases pan-lactylation (Pan-Kla) and H3K18la at the NEAT1 promoter, suppresses NEAT1 transcription, and drives exhaustion programs 115. Lactate can also accelerate terminal differentiation through crosstalk between lactylation and protein homeostasis. It induces Pan-Kla enrichment at the *Usp7* promoter and upregulates USP7, which then deubiquitinates and stabilizes cytochrome c oxidase assembly factor CMC1, rendering CD8⁺ T cells more prone to enter terminal exhaustion 116.

Thus, in a tumor microenvironment enriched in lactate, lactylation converts local metabolic pressure into a mutually reinforcing immunosuppressive network spanning tumor cells, myeloid cells, and T cells, while further promoting terminal exhaustion and mitochondrial dysfunction in CD8⁺ T cells.

#### 3.3.4 ADP-ribosylation

ADP-ribosylation is a modification in which ADP-ribosyltransferases transfer ADP-ribose groups from NAD⁺ onto substrate proteins. It can occur as mono-ADP-ribosylation (MARylation) or poly-ADP-ribosylation (PARylation) 117. In tumor cells, poly(ADP-ribose) polymerase 1 (PARP1) mediates PARylation of limb expression 1-like protein (LIX1L), increases its stability, and promotes CD36-mediated lipid uptake. This remodels a protumorigenic microenvironment and is consistent with impaired maintenance of CD8⁺ T-cell effector function and deviation toward exhaustion 35. This suggests that ADP-ribosylation may link tumor-cell metabolic remodeling to CD8⁺ Tex-associated changes in the immune microenvironment. However, direct evidence for T cell-intrinsic regulation of CD8⁺ Tex programs by ADP-ribosylation remains limited.

Taken together, O-GlcNAcylation, succinylation, lactylation, and ADP-ribosylation involve different metabolic substrates and cellular processes, yet they converge in CD8⁺ Tex to sense, transmit, and inscribe metabolic stress into persistent exhaustion programs, thereby driving sustained exhaustion and progression toward terminal states.

### 3.4 Epigenetic reprogramming

**Figure 6:**
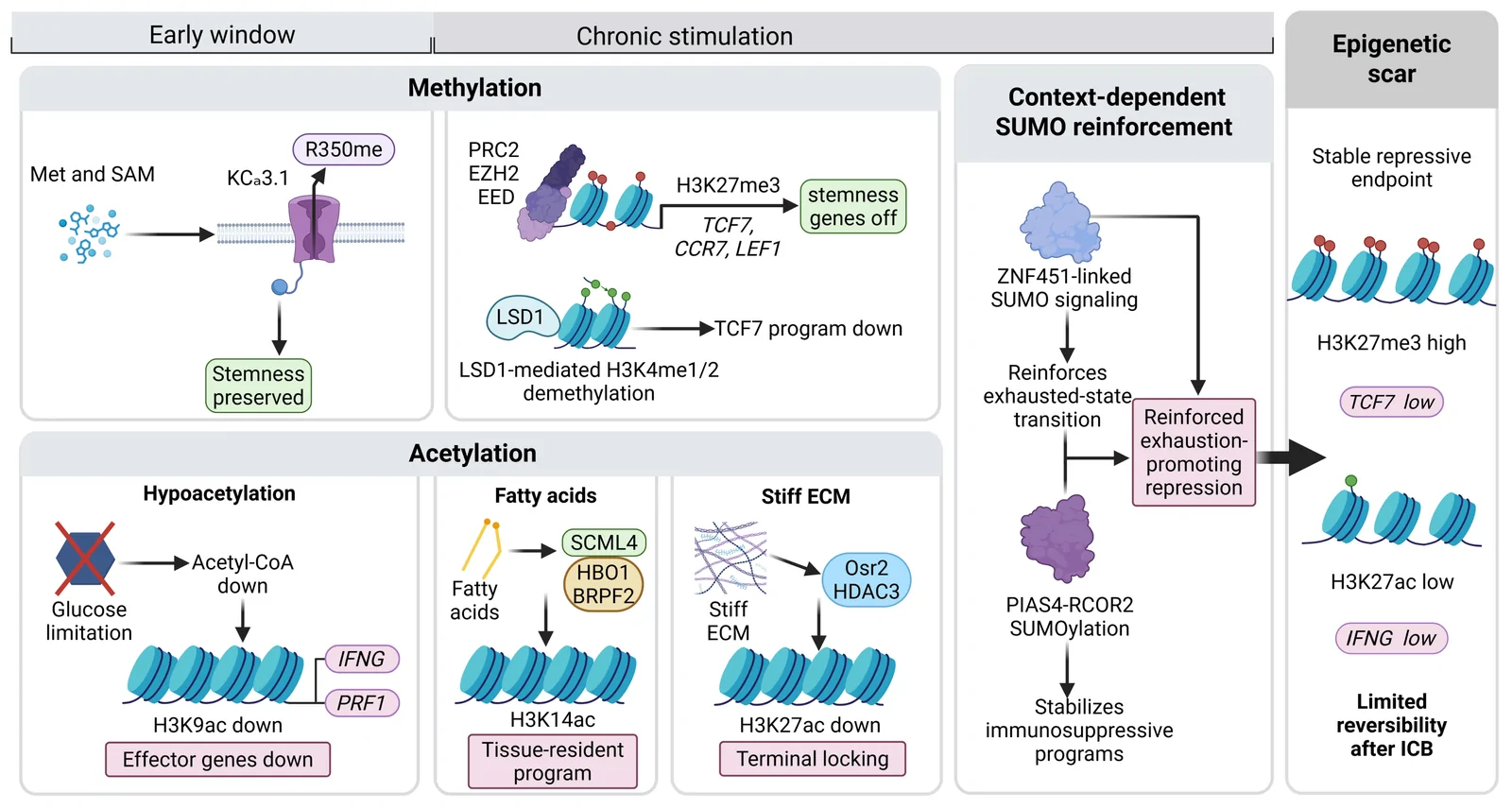
Methylation, acetylation, and SUMOylation drive epigenetic reprogramming in CD8⁺ T-cell exhaustion. Upper left, early window: methionine/S-adenosylmethionine (Met/SAM) supply sustains KCa3.1 R350 methylation and preserves stemness programs. Upper middle, chronic stimulation stage: the polycomb repressive complex 2 (PRC2)-EZH2-EED axis deposits H3K27me3 and suppresses stemness-associated genes, whereas lysine-specific demethylase 1 (LSD1) removes H3K4me1/2 and further attenuates stemness programs. Lower section: aberrant acetylation inscribes metabolic and microenvironmental stress into chromatin states. Glucose restriction lowers acetyl-CoA and H3K9ac, thereby suppressing effector genes. The fatty acid-associated SCML4-HBO1-BRPF2 axis sustains H3K14ac and supports tissue-resident programs in CD8⁺ T cells. A stiff extracellular matrix (ECM) decreases H3K27ac through the Osr2-HDAC3 axis and promotes terminal locking. Middle right: SUMOylation further reinforces inhibitory epigenetic programs under chronic stimulation. Far right: these changes are ultimately deposited as epigenetic scars that show only limited reversibility after ICB.

Mechanisms at the levels of signal transduction, protein homeostasis, and metabolic stress still do not fully explain why CD8⁺ Tex ultimately enter a relatively stable and difficult-to-reverse lineage state. The crucial issue is whether the upstream abnormalities can be further inscribed into chromatin organization, enhancer activity, and transcriptional regulatory complexes so that dynamic responses become fixed as stable epigenetic programs. At this layer, methylation, acetylation, and SUMOylation progressively close stemness-associated programs, maintain exhaustion-related transcriptional networks, and ultimately drive CD8⁺ T cells into a terminally exhausted state (**Fig. 6**).

#### 3.4.1 Methylation

Methylation can occur on histone and non-histone lysine or arginine residues, generating mono-, di-, or trimethylation states. These marks are written mainly by protein arginine methyltransferases (PRMTs) and SET-domain-containing methyltransferases and are erased by lysine-specific demethylase 1 (LSD1) and the lysine demethylase (KDM) family 118. By creating cumulative epigenetic marks, methylation influences both the retention of CD8⁺ T-cell plasticity and the fixation of the exhausted lineage.

This role is first evident in the early activation phase, when T cells are highly sensitive to methyl-donor availability. Within approximately 30 minutes of initial activation, CD8⁺ T cells become acutely dependent on methionine (Met)/S-adenosylmethionine (SAM) supply. When Met/SAM is sufficient, dimethylation of KCa3.1 at Arg350 is maintained, excessive Ca²⁺ influx and NFAT1 nuclear translocation are restrained, and initiation of the exhaustion program is suppressed. By contrast, even transient methionine insufficiency can bias cells toward terminal exhaustion 119. Within the TME, this early deviation can be further amplified by nutrient competition. Tumor cells competitively consume methionine, leading to reduced H3K79me2 in T cells and impaired STAT5 function, whereas methionine supplementation can at least partially restore this axis and improve the exhausted state 120.

During chronic stimulation, methylation further pushes CD8⁺ T cells from reversible suppression toward lineage fixation by silencing stemness-associated genes and maintaining exhaustion-related transcriptional programs. In specific models, increased activity of polycomb repressive complex 2 (PRC2) deposits H3K27me3 at stemness-associated loci such as *Tcf7* and drives the Tpex-to-Tex^term^ transition 121. PRC2 can also suppress C-X-C motif chemokine ligand 10 (CXCL10) through H3K27me3, limit recruitment of CXCR3⁺ CD8⁺ T cells, reduce peripheral effector T-cell replenishment, and thereby reinforce the immunosuppressive microenvironment 122. As the counterpart to methyltransferase activity, demethylation also determines whether this state can be reversed. LSD1 removes H3K4me1/2, antagonizes TCF-1-dependent maintenance programs, silences stemness networks, and promotes terminal exhaustion 123. In PD-1⁺ CD8⁺ T cells, nuclear phosphorylated LSD1 co-localizes with eomesodermin (EOMES) and regulates its modification state, thereby maintaining EOMES-associated exhaustion-like transcriptional output 124. In addition, the macrophage IRG1 (ACOD1)-itaconate axis promotes H3K4me3 at the *EOMES* promoter, increases PD-1/TIM-3, and drives exhaustion 125. Oxidative stress can also induce SET7/9-mediated methylation of sirtuin 1 (SIRT1) and block its secretion, promoting accumulation of PD-L1⁺ M2-like TAMs and thereby deepening CD8⁺ Tex 126.

Thus, methylation drives CD8⁺ T cells from functional suppression toward epigenetic lineage fixation through three coordinated processes: early restriction of methyl-donor availability, chronic silencing of stemness-associated genes, and maintenance of exhaustion-related transcriptional programs.

#### 3.4.2 Acetylation

Acetylation mainly occurs on the epsilon-amino group of lysine residues. It is written by lysine acetyltransferases (KATs), erased by histone deacetylases (HDACs), and read by bromodomain-containing proteins, thereby regulating chromatin accessibility, assembly of transcriptional complexes, and gene-expression programs 127. During the formation of CD8⁺ Tex, acetylation integrates metabolic substrate availability, tissue stress, and tumor-derived inhibitory signals into relatively stable epigenetic states.

One manifestation of this role is the sustained maintenance of exhaustion-associated enhancer activity. Enhancers or super-enhancers adjacent to exhaustion-associated genes such as *Pdcd1*, *Havcr2*, and *Tox* are frequently enriched for H3K27ac, often together with H3K4me3, which is a recurrent epigenetic feature of CD8⁺ Tex 37. In human studies, elderly patients with diffuse large B-cell lymphoma (DLBCL) more often harbor abnormalities in regulators such as TET2 and CREBBP. These changes are accompanied by reduced enhancer activity, decreased histone acetylation, reduced major histocompatibility complex class I (MHC-I), fewer naive T cells, and increased CD8⁺ Tex 128. Such observations suggest that acetylation does not merely affect isolated loci, but participates in defining the enhancer landscape characteristic of CD8⁺ Tex.

Mechanistically, acetylation directly inscribes nutrient restriction and altered metabolic pathways into chromatin remodeling. Prolonged glucose limitation reduces acetyl-CoA production, lowers H3K9/14ac and H3K27ac, compresses chromatin accessibility at effector-gene loci, and leads to exhaustion-like functional decline in CD8⁺ T cells 129. Under chronic antigenic stimulation, nuclear acetyl-CoA becomes more dependent on ATP-citrate lyase (ACLY), and redistribution of H3K27ac, H3K9ac, and H4ac mediated by KAT2A promotes the transition from Tpex to Tex^term^ 130. In contrast, fatty-acid metabolism can limit terminal exhaustion by maintaining expression of SCM polycomb group protein-like 4 (SCML4), increasing H3K14ac, and enhancing chromatin accessibility 131.

In addition to substrate availability, mechanical cues within the TME and tumor-derived suppressive signals can further consolidate exhaustion through acetylation. A stiff extracellular matrix activates an odd-skipped related transcription factor 2 (Osr2) axis that recruits HDAC3 and decreases H3K27ac at cytotoxic gene loci such as* Prf1* and *Ifng*, thereby suppressing cytotoxic programs and promoting Tex^term^ differentiation 132. In tumor cells, casein kinase 2 β (CK2B) progressively increases along the Tpex-to-Tex^term^ trajectory and upregulates HDAC8, which suppresses the T-bet program by reducing H3K27ac at the *TBX21* promoter and thereby promotes a PD-1⁺ TIM-3⁺ terminally exhausted state 133. Tumor-cell acetylation abnormalities may also indirectly shape suppressive niches by altering secretory programs. Aldolase B (ALDOB) translocates into the nucleus and interacts with KAT2A, thereby decreasing H3K9ac at the *TGFB1* promoter and suppressing *TGFB1* transcription. Conversely, ALDOB deficiency increases TGF-β expression and promotes an exhaustion-like phenotype in tumor-infiltrating CD8⁺ T cells 134. Beyond histones, acetylation of non-histone substrates can reshape tumor metabolism and immunogenicity. Under chemotherapy stress, Tat-interactive protein 60 kDa (TIP60) mediates acetylation of glycerol-3-phosphate acyltransferase 3 (GPAT3) at K316, enhances lipid synthesis and lipid-droplet accumulation, and is accompanied by an increased PD-1⁺ TIM-3⁺ exhausted-like population 135. Importantly, although HDAC inhibitors can enhance effector-molecule release by activated CD8⁺ T cells in vitro, they may also produce co-existing effector-like and exhausted-like populations as well as increased myeloid and regulatory T-cell components in vivo, highlighting strong state dependence and timing dependence 136.

Overall, acetylation promotes a more stable terminally exhausted state in CD8⁺ T cells by reshaping enhancer activity and chromatin accessibility, thereby integrating substrate availability, mechanical stress, and tumor-derived inhibitory signals into exhaustion-associated transcriptional programs.

#### 3.4.3 SUMOylation

SUMOylation is a reversible modification in which small ubiquitin-like modifier (SUMO) proteins are conjugated to lysine residues on substrates. It affects gene-expression programs by modulating protein-protein interactions, subcellular localization, and the stability of nuclear transcriptional complexes 137. More broadly, SUMOylation is often associated with transcriptional repression. It can facilitate recruitment of HDAC-containing repressor complexes by modifying transcription factors and co-repressors and can also participate in regulating enhancer acetylation marks and chromatin states 138. At present, however, direct evidence that SUMOylation mediates epigenetic fixation in CD8⁺ Tex remains limited. It is better regarded as a reinforcement mechanism for inhibitory epigenetic programs than as a simple regulator of protein homeostasis.

Available evidence indicates that hypoxia can upregulate REST corepressor 2 (RCOR2) through hypoxia-inducible factor-1α (HIF-1α) and promote PIAS4-mediated SUMO2 modification of RCOR2 at Lys60. This suppresses RCOR2 ubiquitin-dependent degradation and stabilizes it in the nucleus. Stable RCOR2 then enhances transcription of leukemia inhibitory factor (LIF), drives M2-like polarization of TAMs, and promotes accumulation of CD8⁺ Tex 139. These findings indicate that SUMOylation can contribute to maintenance and reinforcement of inhibitory epigenetic programs by stabilizing nuclear transcriptional corepressor networks. Single-cell analyses have also implicated the SUMO E3 ligase ZNF451 in transitions of the CD8⁺ Tex state, although its direct link to epigenetic fixation remains to be defined 140.

Taken together, methylation, acetylation, and SUMOylation act on different substrates and through different biochemical mechanisms, yet they converge on a shared outcome: they convert the earlier layers of altered signaling, protein homeostasis, and metabolic stress responses into stable epigenetic programs.

In summary, CD8⁺ T-cell exhaustion is a progressive process shaped cooperatively by multiple PTM classes across different layers. This view suggests that intervention at PTM nodes should move beyond isolated target blockade and toward combination strategies guided by mechanistic layering, temporal matching, and complementary modes of action.

## 4. Therapeutic opportunities and rational combination strategies to alleviate CD8⁺ Tex

**Figure 7:**
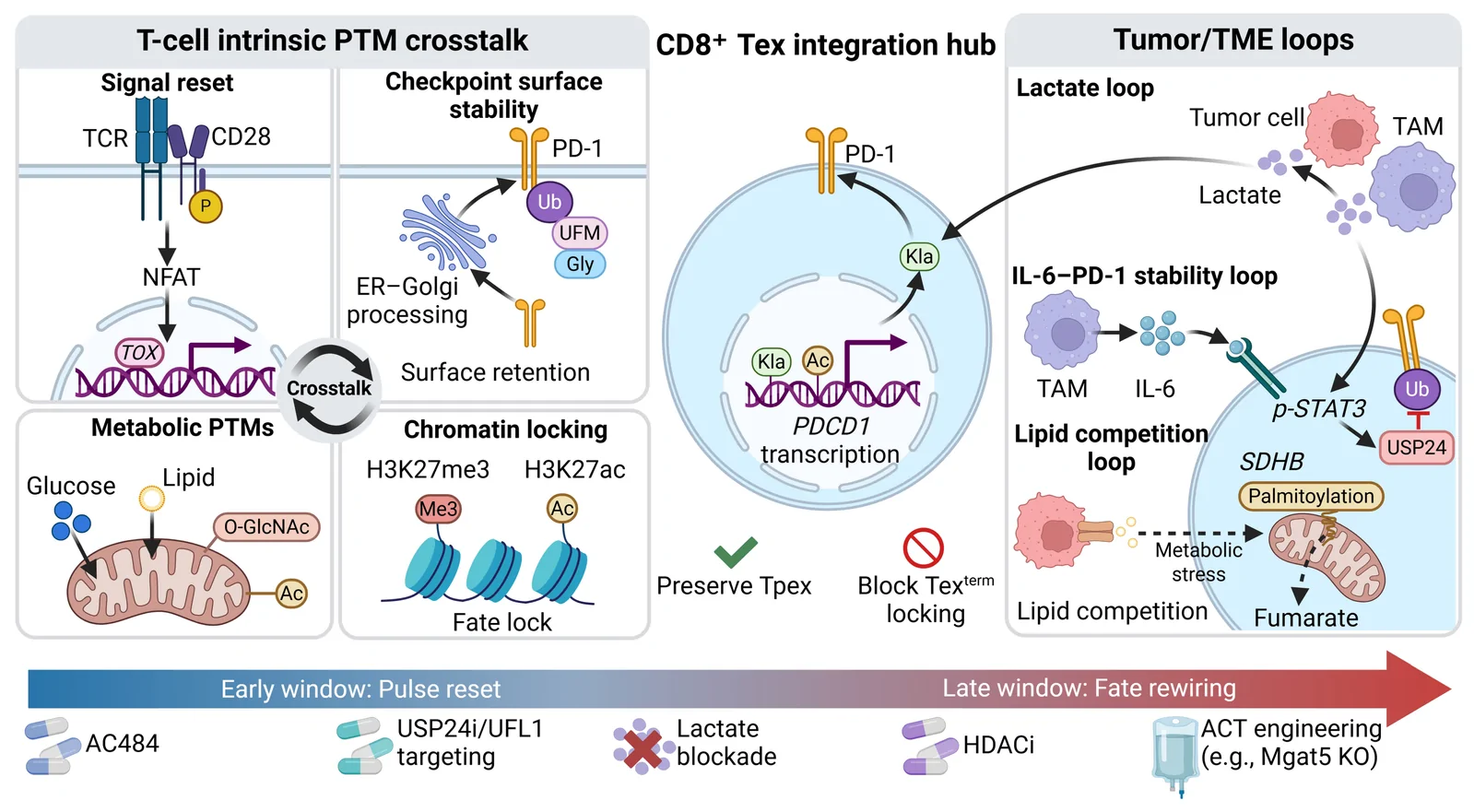
An integrated hub formed by T-cell-intrinsic PTM crosstalk and tumor/TME feedback circuits suggests stage-specific intervention strategies for CD8⁺ Tex. The left panel depicts the intrinsic PTM network in T cells: phosphorylation resets signaling thresholds; glycosylation, UFMylation, and ubiquitination regulate PD-1 surface stability; metabolism-related PTMs reshape mitochondrial states; and chromatin-related PTMs promote fate stabilization. The central hub highlights the joint nuclear control of *PDCD1* transcription by lactylation and acetylation and identifies preserving Tpex while blocking Tex^term^ locking as a key therapeutic objective. The right panel shows tumor- and TME-derived feedback loops, including a lactate circuit, an IL-6-p-STAT3-USP24-mediated PD-1 stabilization loop, and lipid competition-driven metabolic stress. The lower panel proposes two intervention windows along disease progression: an early “pulse-reset” window that focuses on correcting signaling thresholds and checkpoint homeostasis, and a late “fate-rewiring” window that focuses on releasing epigenetic constraints and integrating ACT-based engineering strategies.

Increasing evidence indicates that CD8⁺ T-cell exhaustion is not driven by a single modification event. Rather, it reflects a dynamic process jointly shaped by T-cell-intrinsic PTM crosstalk networks and tumor- or TME-derived extrinsic feedback loops. By altering signal transduction, protein homeostasis, metabolic stress responses, and epigenetic reprogramming, diverse PTMs influence the differentiation trajectory from Tpex to Tex^term^, and therefore offer opportunities to enhance ICB, ACT, radiotherapy, and chemotherapy-based combinations (**Fig. 7**). Representative PTM-oriented intervention strategies aimed at alleviating CD8⁺ T-cell exhaustion are summarized in **Table 1**.

### 4.1 Phosphorylation

Because phosphorylation encodes proximal TCR/CD3ζ immunoreceptor tyrosine-based activation motif (ITAM) signaling as well as downstream co-stimulatory and cytokine receptor signals such as JAK-STAT and AKT-mTOR, it can influence both initiation and progression of exhaustion by resetting activation thresholds and the duration of signal transmission. Among PTM classes, phosphorylation related pathways are currently among the most tractable therapeutic entry points, although their effects remain highly context dependent.

Phosphorylation networks can reduce extrinsic inhibitory input by suppressing the generation or upregulation of inhibitory receptors and ligands along the PD-1/PD-L1 axis. In T cells, the SRPK1 inhibitor SPHINX31 blocks phosphorylation of serine/arginine-rich splicing factor 1 (SRSF1) and functionally achieves an exhaustion-relieving effect similar to PD-1 blockade 141. Under acidic tumor conditions, NaHCO3 buffering combined with the eukaryotic translation initiation factor 4A (eIF4A) inhibitor silvestrol reduces STAT1 phosphorylation and lowers PD-L1 expression in tumor cells 66. Tumor cells also harbor druggable upstream transcriptional nodes. Asiaticoside suppresses SMAD2/3 phosphorylation and ultimately reduces PD-1, TIM-3, and LAG-3 expression on CD8⁺ T cells 142. Ginsenoside Rg5 inhibits the NFKB2/STAT2 axis and downregulates PD-L1 143. Curcumin suppresses PTPN22, restores CBL-mediated K48-linked ubiquitination and degradation of PD-L1, and decreases PD-L1 stability 57. Conventional treatments may also dampen checkpoint supply by interfering with phosphorylation-related pathways. ISP-I combined with temozolomide (TMZ) downregulates PD-L1 through remodeling of GSK-3β/β-catenin-associated phosphorylation signaling 144, and rivaroxaban, a factor Xa (FXa) inhibitor, also reduces STAT2 phosphorylation together with PD-L1 expression 145.

In addition to reducing inhibitory input, phosphorylation-targeted interventions can delay Tex^term^ formation by resetting T-cell-intrinsic phosphorylation hierarchies spanning the proximal TCR network, cytokine receptor pathways, and nuclear programs. ATP depletion under chronic stimulation preferentially damages distal ITAM phosphorylation and thereby promotes exhaustion, suggesting that receptor engineering to reset ITAM phosphorylation hierarchies may improve ICB or ACT efficacy 146. The dual PTPN2/PTPN1 inhibitor AC484 lowers exhaustion by blocking dephosphorylation along the JAK-STAT axis and has entered a phase I clinical trial (NCT04777994) 49. The allosteric PTP1B inhibitor MSI-1436 reduces the proportion of PD-1⁺ TIM-3^hi^ CD8⁺ Tex in tumors by enhancing STAT5 Tyr694 phosphorylation 147. The selective inhibitor nPKC-θi2 blocks nuclear translocation of protein kinase C-θ (PKC-θ), interferes with exhaustion-associated nuclear serine/threonine phosphorylation programs, and relieves Tex 148. Inflammatory STAT-maintenance circuits can also be pharmacologically corrected. Low-dose continuous PF-06651600, a JAK3 inhibitor, reduces terminally exhausted phenotypes by suppressing persistent IL-2R-JAK3-STAT5 phosphorylation 149. Tumor-derived Epstein-Barr virus-induced gene 3 (EBI3) promotes STAT4 phosphorylation and upregulates IL10/CCL5 to induce CD8⁺ Tex, whereas the antagonistic peptide VYLHWHD competitively binds EBI3 and reverses the induced phenotype 150. An aqueous extract of *Euphorbia helioscopia* L. attenuates CD8⁺ Tex in inflammation-driven tumors by lowering STAT3 phosphorylation 151. Some interventions act even further downstream at nuclear and translational phosphorylation outputs. The aurora kinase B (AURKB) inhibitor AZD1152 reduces H3K9me3-S10ph-associated marks and decreases CD8⁺ Tex 152. Perioperative low-dose sirolimus suppresses phosphorylation of ribosomal protein S6 (rpS6), thereby mitigating surgery-induced CD8⁺ Tex 153. MEK or MAPK13 inhibition regulates phosphorylation of the RNAPII-CTD and TCF-1 and helps maintain TCF-1⁺ Tpex cells 26, 154.

Beyond T-cell-intrinsic pathways, phosphorylation-directed interventions can alleviate CD8⁺ Tex indirectly by remodeling tumor-cell and myeloid-cell programs. The GSK3α/β inhibitor SB415286 attenuates exhaustion-like phenotypes by interfering with the GSK3β Ser9 phosphorylation axis 54. The MERTK inhibitor MRX-2843 suppresses receptor tyrosine phosphorylation and alleviates Tex through a dendritic cell-T-cell axis 155. TCP-1-targeted liposomal delivery of parthenolide (PTL) induces phosphorylation-dependent necroptosis in tumor cells and secondarily improves CD8⁺ Tex 156. Licochalcone B remodels the Tex landscape by suppressing phosphorylation of ERK5 and NF-κB p65 157. At the level of herbal formulations, Zuojin capsules improve Tex by inhibiting phosphorylation-dependent activation of mTOR, eIF4E, and p70S6K 158, whereas Xiaoshui Formula suppresses TAM M2 polarization through inhibition of NF-κB p65 phosphorylation and thereby alleviates CD8⁺ Tex 159.

Correction of phosphorylation circuits related to hypoxia and metabolic stress represents another strategy for alleviating CD8⁺ Tex through improvement of the TME and mitochondrial homeostasis. Hypoxic TME drives CD8⁺ T cells toward PD-1^hi^ terminal exhaustion. *Escherichia coli* Nissle 1917 carrying gold-palladium (Au-Pd) nanocatalysts reduces hypoxia-associated exhaustion by suppressing pCREB signaling and synergizes with stereotactic body radiotherapy (SBRT) and anti-PD-L1 treatment 160. Ultrasound-targeted microbubble cavitation (UTMC) induces phosphorylation of endothelial nitric oxide synthase (eNOS) at Ser1177 and Ser633, alleviates hypoxia, reduces CD8⁺ Tex, and can further enhance anti-PD-L1 efficacy 161. Mitochondrial metabolic adaptation is another point of leverage. Diisopropylamine dichloroacetate (DADA) inhibits phosphorylation of the pyruvate dehydrogenase complex (PDC), enhances OXPHOS and mitochondrial fitness, and suppresses terminal exhaustion 162. Under fractionated radiotherapy, the combination of metformin and 2-deoxyglucose (2-DG) liposomes suppresses incomplete mitochondrial outer membrane permeabilization (iMOMP)-integrated stress response (ISR) adaptation and reduces eIF2α phosphorylation, thereby attenuating radiotherapy-induced adaptive PD-L1 upregulation 163.

During ACT manufacturing and engineering, the ex vivo time window provides a more controllable context in which phosphorylation circuits can be preset to improve the Tpex-like features and persistence of reinfused cells. CAR-T cells have been shown to express vascular endothelial growth factor receptor 2 (VEGFR2), suggesting that vascular endothelial growth factor (VEGF)-VEGFR2 signaling may act directly on CAR-T cells rather than being confined to its conventional anti-angiogenic context. Short-term pretreatment with axitinib, a small-molecule VEGFR inhibitor, suppresses VEGFR2 phosphorylation in CAR-T cells and reduces degradation-associated phosphorylation of β-catenin at Ser33/Ser37/Thr41, thereby stabilizing β-catenin, activating Wnt/β-catenin signaling, and alleviating CAR-T cell terminal differentiation and exhaustion 164. Engineered expression of the interleukin-21 receptor (IL-21R) maintains key JAK-STAT phosphorylation signals in the absence of exogenous IL-21, enhances proliferation and cytotoxicity, and reduces inhibitory receptor expression 165. Similarly, interleukin-17A (IL-17A) has been associated with restoration of AKT phosphorylation and a reduction in PD-1⁺ TIM-3⁺ CD8⁺ Tex 166. Relief of dephosphorylation-mediated repression can also be engineered. CRISPR-mediated deletion of SHP-1 enhances cytotoxic output and shows stronger antitumor efficacy when combined with simvastatin 167. Engineering viral tyrosine kinase-interacting protein (TIP) triggers STAT5 phosphorylation, sustains survival and effector function in the absence of IL-2, and suppresses exhaustion differentiation 168. At the mitochondrial level, forkhead box P3 (FOXP3) engineering reduces phosphorylation of DRP1 at Ser616, suppresses mitochondrial fission, rewires metabolism, and ultimately mitigates CD8⁺ Tex 169.

Importantly, phosphorylation-targeted therapy is not inherently equivalent to ICB sensitization. The net effect depends on the functional position of the target as well as timing and dosing intensity. Excessive anabolic signaling can sustain high AKT-mTOR-S6 phosphorylation and accelerate overstimulation and exhaustion-like differentiation of CD8⁺ T cells 170. Ex vivo analyses of patient-derived cells show that dasatinib simultaneously suppresses TCR and STAT5 phosphorylation and has differential effects in TIM-3⁺ T-cell subsets, suggesting marked subset dependence of Src-family kinase-related interventions 171. By contrast, intermittent interleukin-2-inducible T-cell kinase (ITK) inhibition can reactivate exhausted cytotoxic T lymphocytes in multiple ICB-resistant solid-tumor models and enhance ICB efficacy through remodeling of proximal TCR phosphorylation 172. Yet inhibition of protein kinase D (PKD) suppresses AKT phosphorylation in T cells and antagonizes anti-PD-1 therapy 173. Tumor-derived extracellular vesicles can also promote exhaustion through non-canonical pathways. For example, exosomal lncRNA HDAC2-AS2 suppresses cytoplasmic cyclin-dependent kinase 9 (CDK9) in CD8⁺ T cells, reduces p38 phosphorylation, increases PD-1 expression, and aggravates exhaustion 174.

Thus, the key to phosphorylation-directed intervention is not to globally enhance or suppress phosphorylation, but to select targets, delivery modes, and combinations that reset activation thresholds, prevent chronic stimulation from being converted into transcriptional or metabolic burden, block TME-derived amplification of inhibitory phosphorylation, and reduce the supply of inhibitory ligands from tumor cells.

### 4.2 Ubiquitination

Compared with phosphorylation, ubiquitination more directly controls the half-life and cell-surface abundance of inhibitory receptors or ligands, cytokine receptors, and proteins involved in mitochondrial quality control, thereby influencing whether suppressive load can be durably maintained. Because the ubiquitin-proteasome system (UPS) is broadly required for cell survival and stress responses, strategies to alleviate CD8⁺ Tex are better suited to targeting specific DUBs or E3 ligases than to globally inhibiting the UPS.

Targeting selected DUBs and E3 ligases can reduce PD-1 stability in T cells while also diminishing PD-L1 supply from tumors or the microenvironment, thereby reducing sustained suppressive input. USP5 has been identified as a deubiquitinase for PD-1, and its inhibitor EOAI3402143 promotes PD-1 degradation 28. USP24 also deubiquitinates and stabilizes PD-1, whereas USP24-i-101 reduces PD-1 stability and decreases PD-1⁺ TIM-3⁺ CD8⁺ Tex 70. OTUD3 stabilizes PD-L1 through deubiquitination, and the antiallergic drug rupatadine has been reported to inhibit OTUD3 and promote PD-L1 degradation 72. The fusion peptide UM-6 blocks N-glycosylation of PD-L1 and facilitates its ubiquitin-mediated degradation, thereby attenuating PD-1-associated CD8⁺ Tex 175. Microenvironment-driven UPS loops can also be interrupted. A neutralizing antibody against IL-1R2 blocks the ICD-IL1R2-YY1 UPS axis, lowers tumor-cell PD-L1, and enhances anti-PD-1 efficacy 74.

Ubiquitin-dependent regulation can also restore upstream signaling support required for T-cell activation by resetting the homeostasis of cytokine receptors and alternative inhibitory axes. The MARCH5 inhibitor pitavastatin calcium suppresses K27-linked ubiquitination and lysosomal degradation of the common γ-chain (γc), thereby strengthening cytokine signaling and improving exhaustion-associated phenotypes 176. On the bypass-suppression side, USP8 inhibition lowers the steady-state level of TGF-β receptor II (TβRII), alleviates extracellular vesicle-mediated TGF-β signaling, and improves CD8⁺ T-cell function 177.

At the Tex^term^ stage, deficient mitochondrial function together with ROS accumulation and reduced ATP supply forms a major metabolic bottleneck, and the UPS-mitochondrial quality-control axis provides an entry point for metabolic rescue. T-cell-specific high mobility group box 2 (HMGB2) promotes UPS-dependent degradation of nuclear factor erythroid 2-related factor 2 (NRF2), weakens mitochondrial OXPHOS, and can be counteracted by ellagic acid, which synergizes with anti-PD-1 therapy to reduce terminal exhaustion 178. The USP30 inhibitor ST-539 enhances ubiquitination of mitochondrial proteins and mitophagy, improves mitochondrial adaptation, and decreases Tex^term^ 80.

In ACT settings, ubiquitination also affects stability of stem-like populations through chromatin-associated marks. CRISPR disruption of chromatin regulators such as additional sex combs-like 1 (ASXL1) maintains H2AK119ub1 and stabilizes the TCF-1⁺Ly108⁺ Tpex pool, which can synergize with anti-PD-L1 therapy 179. However, because chromatin regulators are pleiotropic, such strategies require more systematic evaluation of off-target effects and long-term phenotypic consequences.

### 4.3 Glycosylation

Glycosylation-directed interventions may alleviate CD8⁺ Tex and enhance treatment response at three levels: restoration of T-cell function, ex vivo glycoengineering, and optimization of delivery. N-glycosylation defects in T cells are associated with impaired effector function, and compensating for the oligosaccharyltransferase (OST) complex can restore IFN-γ production and alleviate CD8⁺ Tex 92. In ex vivo engineering and delivery settings, glycoengineering provides a more direct and controllable means of product optimization. CRISPR knockout of Mgat5 removes β1,6-GlcNAc-branched N-glycans, reduces exhaustion phenotypes, and enhances cytotoxic activity 97. In addition, site-specific conjugation based on glycosylation sites has enabled αPD-1-(iRGD)_2_ to improve antibody penetration into "cold tumors" and to work in combination with ACT 180.

### 4.4 Palmitoylation

Targeting palmitoylation and its upstream lipid-supply pathways can reduce the burden of terminal exhaustion and strengthen antitumor activity. For example, the competitive peptide TIM-3-Palm-WT blocks TIM-3 palmitoylation, reduces PD-1⁺ TIM-3⁺ TOX⁺ TCF-1^-^ CD8⁺ Tex^term^, and enhances the antitumor killing capacity of TILs, CAR-T cells, and natural killer (NK) cells 30. The fatty acid synthase (FASN) inhibitor C75 reduces STAT3 palmitoylation and activation, thereby decreasing PD-1^hi^ TIM-3⁺/CD39⁺ CD8⁺ Tex^term^ 101.

### 4.5 O-GlcNAcylation

Within immunosuppressive niches, the OGT inhibitor OSMI-1 may attenuate CD8⁺ Tex by weakening WTAP-dependent immunosuppressive signaling; in translational studies, its combination with LOXL2 antagonism further improves anti-PD-1 responses 106. In contrast, D-mannose supplementation represents a more T-cell-intrinsic strategy by enhancing β-catenin O-GlcNAcylation, preserving Tcf7-associated stem-like programs, and limiting CD8⁺ Tex progression 32. These findings suggest that the therapeutic effect of O-GlcNAcylation on CD8⁺ Tex depends on the cellular context and modified substrate rather than simple unidirectional inhibition or enhancement.

### 4.6 Lactylation

Inhibition of lactate-driven histone lactylation, particularly the reduction of key lactylation marks such as H3K18la, may suppress the transcription of exhaustion-associated genes and provide a therapeutic entry point for restoring CD8⁺ T-cell effector function. NUPR1 inhibition reduces H3K18la, increases IFN-γ⁺/GzmB⁺ CD8⁺ T cells, and decreases PD-1⁺ CD8⁺ T cells 113. Recent studies have further expanded the therapeutic relevance of the p300 inhibitor C646, suggesting that, beyond its established role in inhibiting p300/CBP histone acetyltransferase activity, it can also be leveraged for lactylation-associated immune reprogramming. CD8α⁺ nanovesicle-mediated delivery of C646 enables targeted entry into exhausted CD8⁺ T cells, reduces H3K18la enrichment at the *PDCD1* promoter, suppresses *PDCD1* transcription and PD-1 expression, and restores IFN-γ and GZMB expression 181.

### 4.7 ADP-ribosylation

Current studies more strongly support ADP-ribosylation as a microenvironment-remodeling or combination-sensitizing module than as a direct reversal strategy for terminal exhaustion. PARP14 inhibition combined with PD-1 blockade regulates macrophage polarization and induces a quiescent state in cytotoxic CD8⁺ T cells, thereby mitigating terminal exhaustion while preserving functionality 182. Whether this class can reverse terminal exhaustion as monotherapy remains unclear.

### 4.8 Methylation

Methylation-oriented strategies often emphasize short-course intervention and precise stratification so as to avoid long-term disruption of the global epigenetic landscape. Brief methionine supplementation or short-term treatment with the KCa3.1 inhibitor TRAM-34 maintains dimethylation of KCa3.1 Arg350 and suppresses initiation of the CD8⁺ Tex program 119. In dietary-metabolic interventions, α-ketoglutarate supplementation shows marked sex dependence. It decreases H3K27me3 and H3K9me3 in females, but may increase H3K27me3 and remodel exhaustion features in males, suggesting that strict stratification and timing control are required 183.

The PRC2-H3K27me3 axis directly participates in the transcriptional repression and reactivation of exhaustion-associated genes. The PRC2 inhibitor EED226 reduces H3K27me3, relieves the epigenetic repression of memory/naïve-associated genes, and downregulates selected exhaustion-associated programs 121. Within the EZH2-H3K27me3-*CXCL10* axis, the EZH2 inhibitor GSK126 may delay exhaustion progression by relieving the epigenetic repression of *CXCL10* 122. Moreover, short-term tazemetostat treatment during ex vivo expansion preferentially reduces H3K27me3 at the *Tcf7* promoter, expands PD-1⁺ TIM-3^-^ TCF-1⁺ Tpex, and decreases Tex^term^ 184. In contrast, engineering strategies further suggest that EZH2 activity can also be used to precondition T cells against tumor-imposed metabolic stress. In tumor-specific CD8⁺ T cells, expression of the gain-of-function EZH2^Y641F^ mutant enhances PRC2-mediated H3K27me3, improves energy metabolism and cytotoxic function, and attenuates the progression of metabolic stress-associated exhaustion 185. These findings suggest that the therapeutic effects of the EZH2-H3K27me3 axis depend on the intervention window, target gene loci, and exhaustion stage.

Besides H3K27me3, H3K4- and H3K36-related marks also participate in maintenance of stemness, recruitment, and exhaustion control in CD8⁺ T cells. Ibuprofen lowers H3K4me3 enrichment at the *EOMES* promoter and slows progression toward Tex^term^ 125. LSD1 inhibitors such as GSK2879552 and ORY-1001 expand the intratumoral TCF-1⁺ Tpex pool by inhibiting removal of H3K4me1/2 36, 123. Fumarate inhibits KDM5, maintains H3K4me3 at the *Tcf7* promoter, expands Tpex, and suppresses Tex^term^ 186. The KDM4C inhibitor SD70 restores H3K36me3, enhances CD8⁺ T-cell recruitment, alleviates exhaustion, promotes inflammatory conversion of the TME, and shows stronger tumor control when combined with radiotherapy and anti-PD-L1 therapy 187.

### 4.9 Acetylation

Because acetylation directly determines the accessibility of enhancers and promoters and whether core transcriptional networks can be reactivated, it can be used both to loosen exhaustion-associated chromatin states and to suppress bypass exhaustion axes or tumor-derived inhibitory output through non-histone acetylation. The small molecule KI-TOX-A3 selectively disrupts the interaction between TOX and lysine acetyltransferase 7 (KAT7), relieves TOX-mediated suppression of histone acetylation, restores H3K14ac, and thereby relaxes exhaustion-associated chromatin constraints 188. Calcitriol increases H3K27ac at the Cd28 promoter through the vitamin D receptor (VDR) and is associated with downregulation of exhaustion-related genes and enhancement of effector function, suggesting that restoration of co-stimulatory accessibility may improve the exhausted phenotype 189.

Compared with targeting a single acetylation node, HDAC inhibition can more broadly reprogram dysfunction linked to histone and non-histone deacetylation. In ACT settings, the class I HDAC inhibitor entinostat (MS-275) reduces exhaustion-associated output and improves antitumor efficacy through epigenetic reprogramming and correction of the TME 190. At a more specific level, the HDAC3 inhibitor RGFP966 interrupts Osr2-mediated recruitment of HDAC3, relieves repression of cytotoxic genes, and suppresses the exhaustion program 132. Selective HDAC6 inhibitors ACY-1215 and ACY-241 also reduce exhaustion-associated phenotypes 191. Upstream correction of the HDAC axis is likewise possible: CK2 inhibition with CX4945 or HDAC8 inhibition with PCI-34051 restores H3K27ac at the *TBX21* promoter and lowers terminal exhaustion 133. The broad-spectrum HDAC inhibitor SAHA (vorinostat) offers an even wider remodeling range and, in some models, both reverses exhaustion-related microenvironmental programs and preserves memory-like features of infiltrating CD8⁺ T cells while suppressing their transition toward exhaustion 192. Beyond this, targeting upstream transcriptional gates may provide better control. The Helios (IKZF2-encoded) inhibitor BNTX enhances accessibility of stemness-associated chromatin, maintains TCF-1⁺ Tpex, and limits progression from Tpex to Tex^term^ 193.

Correcting acetylation can also directly suppress tumor-derived inhibitory output and provide pharmacodynamic readouts in clinical settings. SAHA increases acetylation of JAK1 at Lys1109 and lowers STAT3-driven transcription of fibrinogen-like protein 1 (FGL1), thereby alleviating exhaustion 194. In tumor cells, the KAT2A inhibitor MB3 lowers H3K9ac at the *TGFB1* promoter, restricts TGF-β output, and improves the CD8⁺ Tex phenotype 134. Clinically, a phase I/II study of the broad-spectrum HDAC inhibitor belinostat combined with PAC (cisplatin, doxorubicin, and cyclophosphamide) detected increased acetylation in peripheral CD3⁺ T cells together with immunophenotypic changes, suggesting that acetylation-targeted treatment can serve as a monitorable module in combination regimens 195.

Microbial metabolites can also affect T-cell stemness by inhibiting HDAC activity and remodeling histone acetylation, although their direction of action is strongly dependent on metabolite species, concentration, and model context. Butyrate derived from *Fusobacterium nucleatum* can suppress PD-1 and alleviate CD8⁺ TIL exhaustion through the HDAC3/8-TBX21 axis 196. In contrast, in a solid-tumor ROR1-CAR-T organ-on-chip model, propionate and butyrate induced PD-1 and TIM-3 and weakened infiltration and function, whereas valerate better preserved cytotoxicity together with reduced inhibitory receptors 197. Oral D-mannose can enrich *Faecalibaculum rodentium* and increase propionate and butyrate, thereby enhancing histone acetylation and expanding TCF-1⁺ Tpex.^198^ Supplementation with *Lactobacillus johnsonii* or its metabolite indole-3-propionic acid (IPA) increases H3K27ac at the *Tcf7* super-enhancer and promotes Tpex maintenance 199.

In addition to direct effects on T cells, some microbial-metabolite axes and acetyl-CoA supply pathways may indirectly affect exhaustion by reshaping tumor output or nutrient-driven histone acetylation programs. The efficacy gain observed with capecitabine plus anti-PD-1 has been associated with enrichment of enterotoxigenic Bacteroides fragilis (ETBF) and increased microbiota-derived S1P, which inhibits HDAC1, enhances H3K56ac, and is accompanied by a reduction in exhausted subpopulations 200. Under glucose restriction, acetate supplementation can replenish acetyl-CoA through acetyl-CoA synthetase 2 (ACSS2), restore histone acetylation, and improve IFN-γ production by CD8⁺ T cells 129. By contrast, during exhaustion differentiation, acetyl-CoA supply shifts from acetate dependence to a citrate-dependent route, and the ACLY inhibitor BMS-303141 suppresses Tex^term^ programs and increases Tpex by blocking ACLY-KAT2A-mediated nuclear acetyl-CoA production 130.

### 4.10 SUMOylation

Because SUMOylation mainly contributes to maintenance and reinforcement of inhibitory epigenetic programs in CD8⁺ Tex, current interventions are more appropriately viewed as weakening SUMO-dependent immunosuppressive circuits than as directly reversing terminal stabilization of exhaustion. Preclinical studies show that the SUMO E1 inhibitor TAK-981 (ML-792) lowers SUMO2/3 conjugation in tumor cells and reduces CD8⁺ Tex 201. In TCR-T settings, TAK-981 combined with the DNA methyltransferase inhibitor 5-Aza-2'-dC enhances CD8⁺ TCR-T expansion and persistence while suppressing exhaustion-like differentiation 202. In addition, β-cryptoxanthin inhibits the SUMO E3 ligase ZNF451 and shows synergy with cisplatin in an osteosarcoma resistance model. Because ZNF451 overexpression is associated with increased PD-1 on CD8⁺ T cells, interference with SUMO homeostasis may help weaken maintenance of suppressive phenotypes 140.

Overall, PTM-based therapeutic opportunities should not be understood as single-target solutions. Their rational use depends on disease stage, differentiation state, target position within the PTM network, and compatibility with combination strategies. Interventions that preserve Tpex, prevent progression into Tex^term^, and simultaneously reduce tumor- and TME-derived suppressive reinforcement are likely to be the most promising translational route.

## 5. Conclusions and future perspectives

As discussed throughout this review, formation of CD8⁺ T-cell exhaustion is not driven by a single inhibitory receptor or an isolated signaling abnormality. Instead, it represents a dynamic lineage-evolution process in which persistent antigen stimulation and TME-derived stress are progressively amplified and eventually fixed within PTM networks. PTM biology not only helps explain how CD8⁺ Tex emerge, but also why exhaustion persists and deepens over time. Future work should move beyond cataloguing which modifications are present and instead determine which PTM nodes causally drive exhaustion and at what stage. KLHL6 provides an illustrative example. It promotes TOX polyubiquitination and preserves mitochondrial homeostasis, thereby restricting the transition of precursor exhausted T cells toward terminal exhaustion 41.

At present, however, most evidence still comes from single time points and population-averaged measurements, which makes it difficult to resolve site occupancy, dynamic PTM crosstalk, and spatial heterogeneity across different exhaustion stages. A longitudinal proteomic study tracking antigen-specific CD8⁺ T cells in acute and chronic infection identified more than 180 exhaustion-related proteins and over 900 differentially phosphorylated sites 203. This kind of longitudinal sampling should become a standard framework for future work. Single-cell and spatial multi-omics have further revealed the trajectories and regulators of early versus terminally exhausted T cells 204. Functional genomic screens, including CRISPR interference libraries, have also begun to identify candidate regulatory nodes of exhaustion 205. In addition, proteoform analysis can detect functional protein species defined by specific combinations of modifications and may therefore outperform conventional protein quantification 206. Together, these approaches should enable PTM maps with temporal and spatial resolution and thereby clarify the causal hierarchy among key modification events.

Recent studies also emphasize that post-translational control cannot be separated from protein homeostasis and translational burden in CD8⁺ Tex. A Nature study described a proteotoxic stress response in exhausted T cells (Tex-PSR) that is distinct from canonical stress programs and is characterized by elevated global translation, increased molecular chaperones, protein aggregation, and enhanced autophagy-dependent protein degradation. Perturbing proteostasis itself can push effector T cells toward exhaustion, whereas targeting Tex-PSR-related chaperones improves antitumor immunity 207. Another study showed that RNA-binding protein LARP4 mediates "hypertranslation" that selectively increases translation of nuclear-encoded oxidative phosphorylation genes, disrupts mitochondrial subunit stoichiometry, and causes dysfunction. Deletion of Larp4 reduces translational burden, restores mitochondrial function, and alleviates exhaustion 208. These findings indicate that transcriptional information alone cannot fully reconstruct the true state of CD8⁺ Tex. Future studies should therefore incorporate site-resolved PTM maps, protein homeostasis, and their coupling to translational regulation into the core analytical framework.

Clinical translation of PTM-targeted therapy still faces major challenges. Many modifying enzymes are broadly involved in physiological immune homeostasis and tissue biology, so systemic intervention may cause off-target toxicity, compensatory signaling, and mismatch in treatment timing, as several studies have already cautioned 209, 210. In addition, the biological consequences of a given PTM class are not unidirectional. They depend strongly on the cell type in which the modification occurs, the protein substrate involved, and the differentiation state of the cell. For example, in CD8⁺ T cells, O-GlcNAcylation can maintain *Tcf7* expression and a stem-like program by enhancing O-GlcNAcylation of β-catenin and thus restrain exhaustion differentiation 32, whereas in TAMs it promotes protumor polarization through O-GlcNAcylation of EGR2 and dampens CD8⁺ T-cell antitumor responses 105. Likewise, acetylation can have opposite effects depending on context. Histone acetylation driven by the ACLY-KAT2A complex promotes expression of Tex-associated genes and exhaustion differentiation 130, whereas the SCML4-associated H3K14ac program sustains multifunctional effector properties of tumor-infiltrating CD8⁺ T cells and restrains exhaustion 131. These examples underscore that future PTM-targeted interventions must prioritize cell specificity, stage dependence, and therapeutic windows. Compared with broadly applied monotherapies, more realistic routes may involve precise delivery systems, local intervention, engineered T-cell platforms, and composite biomarkers built from modification-layer information to enable selective remodeling of key PTM networks.

In summary, the value of PTM research in CD8⁺ T-cell exhaustion does not lie in listing ever more modification phenomena. Its real significance lies in establishing a mechanistic framework centered on signal transduction, protein homeostasis, metabolic stress responses, and epigenetic reprogramming. The next key task is to identify PTM nodes that combine causal importance, temporal relevance, spatial specificity, and therapeutic accessibility, and to use them to guide more refined patient stratification and rational combination therapies. Only through this shift can PTM biology move from mechanistic description toward clinical implementation and provide a stronger foundation for overcoming CD8⁺ T-cell exhaustion and improving both the depth and durability of cancer immunotherapy.

## Figures and Tables

**Table 1:** Representative PTM-based intervention strategies currently reported to alleviate CD8⁺ T-cell exhaustion

PTMs	Agents	Targets	Mechanism of action	Combination	Ref
Phosphorylation	SRPK1 inhibitor SPHINX31	SRSF1	Phosphorylation-dependent activation of the splicing factor SRSF1 biases PD-1 mRNA toward the pro-exhaustion membrane isoform (flPD1).	-	141
Phosphorylation	Ginsenoside Rg5	STAT2	Suppresses STAT2 phosphorylation and downregulates PD-L1 expression.	Anti-PD-1 antibody	143
Phosphorylation	Curcumin (PTPN22 inhibitor)	CBL	Blocks CBL Tyr700 dephosphorylation and lowers PD-L1 stability in tumor cells.	Anti-PD-L1 antibody; anti-CTLA-4 antibody	57
Phosphorylation	Factor Xa inhibitor rivaroxaban	STAT2	Downregulates PD-L1 by reducing STAT2 phosphorylation.	Anti-PD-1 antibody	145
Phosphorylation	PTPN inhibitor AC484 (ABBV-CLS-484)	PTPN2/PTPN1	Blocks PTPN2/PTPN1-mediated dephosphorylation, enhances JAK/STAT signaling, lowers the TCR activation threshold, and broadens effector function.	Anti-PD-1 antibody	49
Phosphorylation	PTP1B allosteric inhibitor MSI-1436	STAT5	Inhibits PTP1B activity and enhances STAT5 Tyr694 phosphorylation.	Anti-PD-1 antibody	147
Phosphorylation	PKC-θ inhibitor nPKC-θi2	PKC-θ	Interferes with nuclear PKC-θ-related serine/threonine phosphorylation programs and disrupts the ZEB1/PKC-θ inhibitory complex.	-	148
Phosphorylation	*Euphorbia helioscopia* L.	STAT3	Reduces STAT3 phosphorylation and increases TCF-1 expression.	-	151
Phosphorylation	JAK3 inhibitor PF-06651600	STAT5	Suppresses IL-2R downstream STAT5 phosphorylation and weakens chronic cytokine signaling.	-	149
Phosphorylation	AURKB inhibitor AZD1152	H3K9me3S10ph at the* NCEH1* promoter	Reduces the H3K9me3S10ph mark at the* NCEH1* promoter, restores H3K9me3/HP1 repression, and lowers cholesterol accumulation.	Chemotherapy (gemcitabine or cisplatin) and anti-PD-1 antibody	152
Phosphorylation	MEK inhibitor	RNAPII CTD	Reduces Ser5/Ser2 phosphorylation of the RNAPII-CTD, suppresses energy-intensive nascent transcription, and alleviates mitochondrial NADH/ROS accumulation.	-	26
Phosphorylation	Zuojin capsules	mTOR, eIF4E, p70S6K	Suppresses phosphorylation-dependent activation of mTOR, eIF4E, and p70S6K and downregulates cyclin-dependent kinase 1 (CDK1), thereby remodeling the immunosuppressive TME.	-	158
Phosphorylation	GSK3 inhibitor SB415286	GSK3β	Blocks M2 TAM-derived CCL23/CCR1-GSK3β phosphorylation signaling in CD8⁺ T cells, reducing CTLA-4, TIGIT, TIM-3, LAG-3, and PD-1 expression.	-	54
Phosphorylation	MERTK inhibitor MRX-2843	MERTK	Inhibits MERTK receptor tyrosine phosphorylation, expands CD8α⁺ dendritic cells, and enhances antigen presentation.	-	155
Phosphorylation	Licochalcone B	ERK5, NF-κB	Breaks an NCAM1-associated immunosuppressive lineage by inhibiting ERK5 and NF-κB p65 phosphorylation and inducing ferroptosis.	-	157
Ubiquitination	USP5 inhibitor EOAI3402143	USP5/PD-1	Inhibits USP5 deubiquitinase activity, promotes K48-linked ubiquitination and degradation of PD-1, and reduces PD-1 expression on CD8⁺ T cells.	Anti-CTLA-4 antibody	28
Ubiquitination	USP24 inhibitor USP24-i-101	USP24/PD-1	Inhibits USP24-mediated deubiquitination and lowers PD-1 protein stability.	Anti-CTLA-4 antibody	70
Ubiquitination	OTUD3 inhibitor rupatadine	OTUD3/PD-L1	Inhibits OTUD3-mediated deubiquitination, promotes K48-linked ubiquitination and degradation of PD-L1, and lowers tumor-cell PD-L1 expression.	Anti-PD-L1 antibody	72
Ubiquitination	MARCH5 inhibitor pitavastatin calcium	γc	Inhibits MARCH5-mediated K27-linked polyubiquitination and lysosomal degradation of γc, thereby upregulating γc and enhancing IL-2/STAT5 signaling.	Anti-PD-1 antibody	176
Ubiquitination	USP8 inhibitor	TβRII	Inhibits deubiquitination of TβRII and lowers its plasma membrane stability, markedly reducing the burden of circulating tumor-derived extracellular vesicles.	Chemotherapy (paclitaxel) and anti-PD-L1 antibody	177
Ubiquitination	Ellagic acid	NRF2	Reduces ubiquitin-proteasome degradation of NRF2, activates the NRF2-TFAM axis, and enhances OXPHOS/ATP supply.	Anti-PD-1 antibody	178
Ubiquitination	USP30 inhibitor ST-539	USP30	Suppresses USP30 transcription and protein expression, thereby relieving PINK1/Parkin pathway-mediated defects in mitophagy.	-	80
Palmitoylation	FASN inhibitor C75	STAT3	Inhibits fatty acid synthase (FASN) and reduces STAT3 palmitoylation and activation.	Anti-PD-1 antibody	101
O-GlcNAcylation	OGT inhibitor OSMI-1	WTAP	Inhibits OGT and OGT-mediated O-GlcNAcylation of WTAP, weakens WTAP stability, and suppresses M2-like TAM differentiation.	-	106
O-GlcNAcylation	D-mannose	β-catenin	Increases UDP-GlcNAc supply, enhances OGT-mediated O-GlcNAcylation of β-catenin, promotes its nuclear translocation, and drives stemness-associated transcriptional programs such as *Tcf7*.	-	32
Lactylation	NUPR1 inhibitor	NUPR1	Reactivates ERK/JNK-MAPK signaling in TAMs, reverses M2-like polarization, and reduces PD-L1/SIRPA-mediated immunosuppressive signaling.	Anti-PD-1 antibody	113
Methylation	KCa3.1 inhibitor TRAM-34	KCa3.1	Maintains dimethylation of KCa3.1 Arg350, restricts excessive Ca²⁺ influx and NFAT1 nuclear translocation, and suppresses initiation of exhaustion programs such as *Pdcd1*/*Lag3*.	Anti-PD-1 antibody	119
Methylation	PRC2 inhibitor EED226; EZH2 inhibitor GSK126; tazemetostat	H3K27me3	Inhibits EZH2/PRC2, lowers H3K27me3, relieves silencing of stemness-associated genes such as *Tcf7*, and expands the TCF-1⁺ Tpex pool.	Anti-PD-1 antibody	121, 122, 184
Methylation	Ibuprofen	H3K4me3 at the *EOMES* promoter	Inhibits the macrophage IRG1/itaconate axis and blocks the itaconate-succinate-H3K4me3-EOMES exhaustion program.	Anti-PD-1 antibody	125
Methylation	LSD1 inhibitors GSK2879552 and ORY-1001	LSD1, H3K4me1/2	Inhibits LSD1-mediated demethylation, increases H3K4me1/2, and expands the intratumoral TCF-1⁺ Tpex pool.	Anti-PD-1 antibody	122
Methylation	Fumarate	KDM5/H3K4me3	Inhibits KDM5-mediated H3K4me3 demethylation, maintains H3K4me3 and TCF-1 expression at the *Tcf7* promoter.	Anti-PD-L1 antibody	186
Methylation	KDM4C inhibitor SD70	KDM4C; H3K36me3 at the *CXCL10* promoter	Increases H3K36me3 enrichment at the *CXCL10* promoter by inhibiting KDM4C, upregulates CXCL10, enhances CD8⁺ T-cell migration and activation, and lowers PD-1/CD39 expression.	Radiotherapy and anti-PD-L1 antibody	187
Acetylation	HDAC6 inhibitors ACY-1215 and ACY-241	HDAC6	Downregulates the GATA3-driven T helper 2 (Th2) transcriptional program, weakens immunosuppressive programs, and increases T-bet.	-	191
Acetylation	HDAC inhibitor SAHA (vorinostat)	HDAC, JAK1	Globally increases acetylation and remodels the exhaustion trajectory; may also weaken the FGL1-LAG3 axis through JAK1 Lys1109 acetylation.	-	192
Acetylation	CK2 inhibitor CX4945; HDAC8 inhibitor PCI-34051	H3K27ac at the *TBX21* promoter	Restores H3K27ac at the *TBX21* promoter and upregulates T-bet.	Anti-PD-1 antibody	133
Acetylation	HDAC3 inhibitor RGFP966	HDAC3/H3K27ac at the *Prf1* and *Ifng* promoters	Blocks Osr2-mediated recruitment of HDAC3, restores H3K27ac at loci including *Prf1* and *Ifng*, and releases epigenetic repression of cytotoxic genes.	-	132
Acetylation	Class I HDAC inhibitor entinostat (MS-275)	HDAC1/3	Inhibits HDAC1/3-mediated histone deacetylation, restores immune-cell function in tumors, and suppresses immunosuppressive pathways driven by increasing tumor burden.	-	190
Acetylation	KAT2A inhibitor MB3	KAT2A/H3K9ac at the *TGFB1* promoter	Inhibits the histone acetyltransferase activity of KAT2A, lowers H3K9ac at the *TGFB1* promoter, and restricts TGF-β output.	-	134
Acetylation	ACLY inhibitor BMS-303141	ACLY	Blocks ACLY-KAT2A-mediated citrate-dependent nuclear acetyl-CoA supply.	Anti-PD-L1 antibody	130

## Abbreviations

ACLY: ATP-citrate lyase
ACT: adoptive cell therapy
AKT: protein kinase B
ATP: adenosine triphosphate
AURKB: aurora kinase B
BATF2: basic leucine zipper ATF-like transcription factor 2
CAR-T: chimeric antigen receptor T cell
CBL: Casitas B-lineage lymphoma proto-oncogene
CCL23: C-C motif chemokine ligand 23
CK2B: casein kinase 2 β
CPT1A: carnitine palmitoyltransferase 1A
CRISPR: clustered regularly interspaced short palindromic repeats
CTLA-4: cytotoxic T-lymphocyte-associated protein 4
CXCL10: C-X-C motif chemokine ligand 10
DRP1: dynamin-related protein 1
DUB(s): deubiquitinase(s)
EBI3: Epstein-Barr virus-induced gene 3
EGR2: early growth response 2
eIF4E: eukaryotic translation initiation factor 4E
EOMES: eomesodermin
ERK: extracellular signal-regulated kinase
EZH2: enhancer of zeste homolog 2
FASN: fatty acid synthase
FGF2: fibroblast growth factor 2
FGL1: fibrinogen-like protein 1
FUT2: fucosyltransferase 2
FUT8: fucosyltransferase 8
GSK-3β: glycogen synthase kinase 3β
GZMK: granzyme K
HAVCR2: hepatitis A virus cellular receptor 2
HDAC(s): histone deacetylase(s)
ICB: immune checkpoint blockade
IFN-γ: interferon-γ
INPP4B: inositol polyphosphate 4-phosphatase type II B
IRF4: interferon regulatory factor 4
ITAM: immunoreceptor tyrosine-based activation motif
ITIM(s): immunoreceptor tyrosine-based inhibitory motif(s)
JAK: Janus kinase
KAT2A: lysine acetyltransferase 2A
KDM: lysine demethylase
KLHL6: kelch-like family member 6
LAG-3: lymphocyte activation gene-3
LAT: linker for activation of T cells
LCK: lymphocyte-specific protein tyrosine kinase
LIX1L: limb expression 1-like protein
LSD1: lysine-specific demethylase 1
MARCH5: membrane-associated RING-CH-type finger 5
MDSC(s): myeloid-derived suppressor cell(s)
MEK: mitogen-activated protein kinase kinase
MERTK: MER proto-oncogene tyrosine kinase
mTOR: mechanistic target of rapamycin
NAD⁺: nicotinamide adenine dinucleotide
NCEH1: neutral cholesterol ester hydrolase 1
NEAT1: nuclear paraspeckle assembly transcript 1
NFAT: nuclear factor of activated T cells
NFAT1: nuclear factor of activated T cells 1
NRF2: nuclear factor erythroid 2-related factor 2
NUPR1: nuclear protein 1
OGT: O-GlcNAc transferase
OST: oligosaccharyltransferase
OTUD3: OTU deubiquitinase 3
Osr2: odd-skipped related transcription factor 2
OXPHOS: oxidative phosphorylation
Pan-Kla: pan-lysine lactylation
PARP1: poly(ADP-ribose) polymerase 1
PARylation: poly-ADP-ribosylation
PD-1: programmed cell death protein 1
PD-L1: programmed death-ligand 1
PD-L2: programmed death-ligand 2
PDCD1: programmed cell death protein 1 gene
PI3K: phosphoinositide 3-kinase
PINK1: PTEN-induced kinase 1
PKC-θ: protein kinase C-θ
PRC2: polycomb repressive complex 2
Prf1: perforin 1
PSGL-1: P-selectin glycoprotein ligand 1
PTM(s): post-translational modification(s)
PTP1B: protein tyrosine phosphatase 1B
PTPN: protein tyrosine phosphatase non-receptor type
PTPN22: protein tyrosine phosphatase non-receptor type 22
Rab37: Ras-related protein Rab-37
RBPJ: recombination signal binding protein for immunoglobulin kappa J region
RCOR2: REST corepressor 2
RNAPII-CTD: RNA polymerase II carboxy-terminal domain
ROS: reactive oxygen species
SAM: S-adenosylmethionine
SCML4: SCM polycomb group protein-like 4
SDHB: succinate dehydrogenase complex subunit B
SHP-1: Src homology region 2 domain-containing phosphatase-1
SIRT7: sirtuin 7
SMAD: SMAD family members
SRPK1: serine/arginine-rich protein-specific kinase 1
SRSF1: serine/arginine-rich splicing factor 1
STAT: signal transducer and activator of transcription
TAM(s): tumor-associated macrophage(s)
TBX21: T-box transcription factor 21
TCF-1: T-cell factor 1
TCF7/Tcf7: T-cell factor 7
TCR: T-cell receptor
TCR-T: T-cell receptor-engineered T cell
Tex: exhausted T cells
Texint: intermediate exhausted T cells
Texterm: terminally exhausted T cells
TGF-β: transforming growth factor-β
TGFB1: transforming growth factor-β1 gene
TIGIT: T-cell immunoreceptor with Ig and ITIM domains
TIL(s): tumor-infiltrating lymphocyte(s)
TIM-3: T-cell immunoglobulin and mucin-domain-containing protein 3
TME: tumor microenvironment
TNF: tumor necrosis factor
TNF-α: tumor necrosis factor-α
TNFRSF1B: tumor necrosis factor receptor superfamily member 1B
TOX: thymocyte selection-associated high mobility group box
TβRII: transforming growth factor-β receptor II
TSH: thyroid-stimulating hormone
UFM1: ubiquitin-fold modifier 1
UPS: ubiquitin-proteasome system
VISTA: V-domain Ig suppressor of T-cell activation
WTAP: Wilms tumor 1-associated protein
YY1: Yin Yang 1
ZAP70: ζ-chain-associated protein kinase 70
ZDHHC5: zinc finger DHHC-type palmitoyltransferase 5
ZNF451: zinc finger protein 451
